# Evaluation of Plant-Based Egg Substitutes in Vegan Muffins: Functional, Structural, and Nutritional Characterization

**DOI:** 10.3390/foods14173012

**Published:** 2025-08-28

**Authors:** Kübra Topaloğlu Günan, Perihan Yolci Ömeroğlu

**Affiliations:** 1 Food Engineering Department, Faculty of Agriculture, Bursa Uludag University, Gorukle Campus, 16059 Bursa, Türkiye; kubratopaloglu@maltepe.edu.tr; 2 Department of Gastronomy and Culinary Arts, Faculty of Fine Arts, Maltepe University, 34857 Istanbul, Türkiye

**Keywords:** muffins, plant-based, egg substitute, vegan

## Abstract

As demand for plant-based bakery products rises, suitable egg substitutes are needed to preserve product quality. This study evaluated the functional, nutritional, and sensory effects of plant-based egg substitutes in vegan muffins, focusing on texture, rheology, nutrition, antioxidants, amino acids, and storage. To address this, muffins were prepared by replacing eggs with chickpea aquafaba, chia gel, flaxseed gel, psyllium husk, ripe banana, and soapwort extract, and each formulation was systematically characterized. Rheological analysis showed that banana and chia gel improved viscoelastic behavior, while soapwort enhanced foaming capacity. Hardness varied between 1266.15 gf in soapwort muffins and 2735.73 gf in chia muffins (*p* < 0.05). Compositional analysis showed that protein content varied between 5.24 and 8.54 g/100 g, whereas psyllium and flaxseed significantly increased dietary fiber levels (1.50 g/100 g; *p* < 0.05). Chia and psyllium also enhanced the total phenolic content and antioxidant bioaccessibility. While the plant-based muffins showed lower levels of essential amino acids, they contained sufficient amounts of arginine and glutamic acid. Sensory scores ranged between 6.00 and 8.50, with banana muffins closest to the control. Principal component analysis highlighted ingredient-specific differences. These findings support the use of plant-based ingredients as functional egg replacements in vegan muffin formulations.

## 1. Introduction

Growing concerns about health, sustainability, and ethical food production have led to a significant shift toward plant-based diets, which aim to reduce reliance on animal-derived ingredients [1]. Consequently, vegetarian and vegan lifestyles have gained increasing popularity worldwide [2]. In response to this trend, researchers and food technologists have focused on developing plant-based alternatives to commonly used animal-based components—especially eggs, which play key functional roles in baked goods such as emulsification, binding, and leavening [3]. Muffins, in particular, depend heavily on eggs for structure, texture, and volume [4]. However, factors such as the high cost of egg production, allergenic proteins such as avidin, and health concerns related to cholesterol have driven interest in egg-free bakery formulations [5]. Replicating the multifunctional roles of eggs with plant-based alternatives remains a substantial formulation challenge [3].

Among the promising candidates for plant-based egg substitution is aquafaba, a viscous liquid by-product of cooking legumes. Rich in proteins and carbohydrates, aquafaba has been shown to possess foaming, emulsifying, and thickening properties [6]. Chickpea aquafaba, in particular, has demonstrated the ability to improve muffin height, volume, and texture, making it suitable for sponge-based applications [7,8,9].

Other common plant-based egg substitutes include flaxseed gel, chia seeds, psyllium husk, soapwort extract (*Saponaria officinalis*), and ripe bananas. Flaxseed is a widely used plant-based egg substitute due to its high dietary fiber content and ability to form a mucilage-like gel upon hydration. This gel improves water retention, emulsification, and foaming capacity, thereby enhancing muffin texture and structure [10]. Similarly, chia seeds exhibit similar properties and are valued for their emulsifying potential and stability in various food systems [11,12]. Psyllium husk is another effective alternative, known for its strong water-binding and gel-forming capabilities. It increases batter viscosity and contributes to a more uniform texture in baked goods [13,14].

Soapwort extract, which is rich in saponins, has been used as a natural foaming and emulsifying agent in bakery and confectionery applications to enhance moisture retention and structural stability [15]. Despite its potential, its use as an egg replacer remains marginally explored, particularly regarding structural and nutritional functionality [15,16].

Ripe bananas, often considered food waste, are another sustainable option. They contain functional compounds such as pectin and maltodextrin, which contribute to foaming, fat-holding capacity, and water retention while reducing batter density. Overripe bananas have been shown to enhance muffin volume, tenderness, and moisture content, making them a promising clean-label substitute [17,18]. Furthermore, bananas contribute sweetness, texture enhancement, and antioxidant activity [7,18].

Although plant-based egg alternatives have received increasing attention, most investigations to date have focused on individual substitutes and isolated parameters. Studies examining chia and flaxseed gels have primarily reported improvements in moisture retention, crumb structure, and sensory acceptability in baked goods [11,12,19,20]. However, few studies have compared multiple substitutes under consistent baking and analytical conditions, limiting cross-comparability and practical application [3,9,21]. In addition, the existing literature predominantly focuses on physical and sensory assessments, frequently neglecting important nutritional parameters such as amino acid composition, antioxidant capacity, and shelf-life stability [7,18]. Furthermore, studies utilizing in vitro digestion models to evaluate antioxidant bioaccessibility—an essential aspect in the development of functional bakery products—remain scarce [16,21].

To address these knowledge gaps, this study aims to develop a scientifically validated and sensory-acceptable vegan muffin by replacing eggs with six widely used plant-based substitutes: aquafaba, chia gel, flaxseed gel, psyllium husk, ripe banana, and soapwort extract. In this study, the term vegan muffin refers to muffins prepared without any animal-derived ingredients, in which eggs are substituted with plant-based proteins and cow’s milk is replaced with almond milk. The research provides a comprehensive evaluation of each substitute’s impact on batter rheology, muffin texture, volume, and moisture retention. Additionally, it assesses nutritional parameters, including protein, fiber, and fat content, amino acid composition, total phenolics, antioxidant capacity, and bioaccessibility following in vitro digestion. Sensory evaluation and a 30-day shelf-life evaluation are also included. Finally, chemometric modeling using PCA and HCA is conducted to generate multidimensional insights. This integrative approach supports the development of sustainable, minimally processed, and nutritionally enhanced bakery products formulated with natural and recognizable ingredients, in line with modern dietary preferences.

## 2. Materials and Methods

### 2.1. Materials

Almond milk, a plant-based milk alternative with 2.3% almond content, was obtained from Danone Tikveşli Gıda ve İçecek San. ve Tic. A.Ş. (Istanbul, Türkiye). According to the product label (Alpro, Ghent, Belgium), it contained 13 kcal energy, 1.1% fat (0.1% saturated), 0.0% carbohydrates and sugars, 0.4% protein, 0.3% fiber, and 0.1% salt per 100 mL. It was fortified with calcium (120 mg) and vitamins D, E, B2, and B12. The formulation included water, almonds, tricalcium phosphate, sea salt, stabilizers (locust bean gum and gellan gum), and sunflower lecithin. Aquafaba was prepared from boiled chickpeas. Chickpeas were soaked at a 1:4 (*w*/*v*) ratio (500 g chickpeas with 2000 mL water) for 8–10 h at 4 °C. After soaking, they were drained and cooked in a pressure cooker (Fissler, Vitavit, 6 L, Idar-Oberstein, Germany) at a 2:3 (*w*/*v*) ratio (500 g chickpeas with 750 g water) for 30 min at 98 °C. After cooking, they were cooled to room temperature (25 ± 2 °C) and stored in a refrigerator (4 ± 1 °C) for 24 h to stabilize the soluble components. The chickpea water was then drained and the chickpeas left to reach room temperature (25 ± 2 °C) before being whipped at high speed (220 rpm) for 10 min using a stand mixer (KitchenAid, Classic, 4.3 L, St. Joseph, MI, USA) to obtain a foamy aquafaba, as described by de Barros Miranda et al. [22]. Banana puree was prepared using a blender (Zwilling Enfinigy, 1.4 L, Solingen, Germany) to blend 100 g of peeled, fully ripened banana (*Musa cavendishii*) at medium speed (level 6) for 1 min. The banana puree was used immediately to prevent enzymatic browning. Hydrocolloid-based binders (chia, psyllium, and flaxseed) were prepared as follows:

• Chia seeds (*Salvia hispanica* L., Yayla, Ankara, Türkiye, origin: Argentina) and psyllium powder (Wefood, İstanbul, Türkiye) were hydrated in distilled water at a 1:5 (*w*/*v*) ratio for 10 min at room temperature (25 ± 2 °C) following previously reported approaches, with slight modifications [11,12,14].
• Flaxseeds (*Plantago ovata* Forssk., Yayla, Ankara, Türkiye, origin:China) were first ground using a spice grinder (Fakir Aromatic, Germany) for 20 s. The ground flaxseeds were then sieved through a stainless steel sieve with a pore size of 500 µm, dispersed in distilled water at a 1:5 (*w*/*v*) ratio, and hydrated for 10 min at room temperature (25 ± 2 °C) to form a mucilage-based gel, intended for use as a plant-derived binding agent.
• Soapwort extract (*Saponaria officinalis* L., original concentration: 1:10 *w*/*v*) was diluted 1:5 (*v*/*v*) in distilled water before use. This dilution ratio was selected based on preliminary trials and the recommendations of Çelik et al. [23], aiming to mimic the foaming capacity of aquafaba while maintaining flavor neutrality.

The remaining ingredients in the recipe, which included wheat flour (Sinangil, Tekirdağ, Türkiye), sunflower oil (Yudum, Balıkesir, Türkiye), granulated sugar (Balküpü, İstanbul, Türkiye), baking powder (Dr. Oetker, İzmir, Türkiye), and vanillin (Dr. Oetker, İzmir, Türkiye), were purchased from a local market in Istanbul. All ingredients were stored in their original packaging under cool and dry conditions (22–24 °C, <50% RH) until use. The banana was stored at room temperature (25 ± 2 °C) and used at full ripeness (peel covered with brown spots, indicating peak sugar development).

### 2.2. Chemicals and Reagents

All chemicals used in the analyses were of analytical or chromatographic grade, unless otherwise specified. For proximate composition analyses, petroleum ether (boiling point 40–60 °C), hydrochloric acid (37%), nitric acid (≥65%, Suprapur), and hydrogen peroxide (30%) were purchased from Merck (Darmstadt, Germany). Enzymes used for total dietary fiber determination—heat-stable α-amylase, protease, and amyloglucosidase—were obtained through a commercial enzymatic kit (Megazyme Total Dietary Fiber Assay Kit, Bray, Ireland). For mineral analysis, Suprapur-grade nitric acid (65%) and hydrogen peroxide (30%) were sourced from Merck (Darmstadt, Germany). Calibration standards for ICP-MS were prepared using a multi-element standard solution (ICP multi-element standard VI, Merck, Darmstadt, Germany) and diluted in 1% HNO_3_ prepared with ultrapure water (resistivity ≥18.2 MΩ·cm, Milli-Q system, Millipore, Burlington, MA, USA). Standard solutions were stored in acid-washed polyethylene vials at 4 °C. High-purity argon (99.99%) was used as the plasma gas. For spectrophotometric determination of total phenolic content (TPC), Folin–Ciocalteu reagent, sodium carbonate, and gallic acid (≥98% purity) were obtained from Merck (Darmstadt, Germany). Total flavonoid content (TFC) was measured using aluminum chloride, sodium nitrite (Merck), and sodium hydroxide (Sigma-Aldrich, St. Louis, MO, USA), with rutin (≥98%) as the reference standard (Acros Organics, Fair Lawn, NJ, USA). Unless otherwise noted, all solvents (ethanol, acetonitrile, etc.) and chemicals were of analytical grade and purchased from Merck (Darmstadt, Germany) or Sigma-Aldrich (St. Louis, MO, USA).

### 2.3. Methods

#### 2.3.1. Muffin Preparation

Muffin production was adapted from the method described by Topkaya and Isik [24], with modifications to incorporate plant-based egg alternatives. The components used in muffin preparation are listed in Table 1. For the control sample (CC), eggs and sugar were mixed at high speed (220 rpm) for 4 min using a stand mixer (Kitchen Aid, Classic 4.3 L, St. Joseph, MI, USA) with a whisk attachment. Sunflower oil and milk (cow milk for CC, almond milk for all other samples) were then added and mixed at medium speed (135 rpm) for 1 min. Subsequently, dry ingredients (wheat flour, baking powder, and vanillin) were incorporated at low speed (95 rpm) for an additional 1 min. Each plant-based substitute (aquafaba, banana, chia gel, flaxseed gel, psyllium gel, or soapwort extract) was used in place of whole eggs at an equivalent weight of 90 g, based on a study reporting comparable functional behavior [25]. The substitution amount for each egg alternative was standardized to 90 g to simulate a consistent replacement ratio commonly used in muffin recipes. This approach aimed to assess the intrinsic functional differences of each substitute under identical conditions, rather than adjusting for compositional disparities in water, protein, or lipid content. We acknowledge that these compositional variations may influence certain functional attributes such as texture or moisture retention; however, our focus in this study was on evaluating their performance under a unified replacement scenario rather than optimizing formulations for each individual substitute. Almond milk was added at 32 g to match the amount of cow milk used in the control formulation.

A total of 40 g of muffin batter was poured into a 12-cup muffin tray (Zenker, Germany), with each compartment measuring 7.0 × 5.6 cm. The muffins were baked in a preheated industrial convection oven (Inoksan, INO-FKE010, Bursa, Türkiye) at 170 ± 2 °C for 16 min using fan mode. All muffins were cooled in the molds at ambient temperature (25 ± 2 °C) for 30 min and then removed. Three independent batches were prepared and analyzed for each formulation.

#### 2.3.2. Muffin Properties

After the muffin samples were removed from the oven, they were first left to cool in the molds at room temperature (25 ± 2 °C) for 30 min and then carefully removed from the molds. Subsequently, for standardization of the physical and structural measurements, the muffins were allowed to rest at room temperature (25 ± 2 °C) for 24 h before analysis. Volume index (VI), symmetry index (SI), uniformity index (UI), texture, color, and scanning electron microscopy (SEM) analysis were conducted. The VI, SI, and UI of the muffin samples were measured using a digital caliper (±0.01 mm accuracy) from three predefined points on the muffin surface (center, left, and right edges) and were evaluated following the AACC 10–91 [26]. Volume index (VI) represents the average height of the muffin and reflects the overall rise. It was calculated using the formula given in Equation (1):

(1) $$VI=\frac{A+B+C}{3}\;(\mathrm{mm})$$

where A is the height at the center of the muffin, and B and C are the heights at the left and right edges, respectively. Symmetry index (SI) evaluates the balance between the left and right sides of the muffin and was calculated using the absolute difference (Equation (2)):

(2) SI=|B−C| (mm)

Lower SI values indicate better symmetry. An SI ≤ 5 mm is considered acceptable, while larger values may indicate uneven baking or heat distribution. Uniformity index (UI) reflects how evenly the muffin has risen across its surface. It was calculated using the mean of the absolute deviations between the center and edges (Equation (3)):

(3) $$UI=\frac{\left|A-B|+|A-C\right|}{2}\;(\mathrm{mm})$$

Lower UI values indicate a more uniformly domed or flat muffin. A UI ≤ 5 mm is typically considered satisfactory, whereas higher values may indicate center collapse or edge overexpansion. All measurements were performed after the muffins had cooled to room temperature (25 ± 2 °C).

The VI, SI, and UI of the muffin samples were evaluated following the AACC 10–91 [26]. Baking loss was calculated by comparing the weight of the muffin batter before baking (wd) with its weight after cooling (wc), as expressed in Equation (4) [27].

(4) $$Baking\;Loss=\frac{wd-wc}{wd}\times 100\;(\%)$$

Analysis of the texture of the muffin samples was conducted using a Stable Micro Systems TAXTPlus texture analyzer. After cooling for 24 h, samples were cut into 2.5 cm cubes [28]. A double compression test was performed to evaluate five key texture parameters: hardness, springiness, cohesiveness, chewiness, and resilience. The test was conducted using a 36 mm cylindrical probe at a speed of 1.0 mm/s and 40% deformation, with a load cell capacity of 5 kg and a trigger force of 5 g. Hardness refers to the force needed to compress the muffin during the first contact, indicating how firm or dense the structure is. Springiness reflects the ability of the muffin to return to its original shape after being compressed, revealing the product’s elasticity. Cohesiveness describes how well the muffin holds together under repeated compression, showing the strength of its internal bonds. Chewiness represents the effort required to prepare the muffin for swallowing, reflecting both structure and elasticity. Resilience refers to how quickly and completely the muffin regains its shape immediately after being compressed [29].

Flow behavior was evaluated through steady shear measurements performed over a shear rate range of 0.01–100 s^−1^ under controlled shear rate mode. The resulting flow curves (shear stress vs. shear rate) were fitted using the Power Law model, given in Equation (5):

(5) $$\tau =K{\dot{\gamma }}^{n}$$

where τ is the shear stress (Pa), K is the consistency index (Pa s*^n^*), *n* is the flow behavior index (dimensionless), and $\dot{\gamma }$ is the shear rate (s^−1^). These parameters were used to evaluate and compare the pseudoplastic characteristics of different batter formulations.

Dynamic oscillatory measurements of the muffin batters were performed using a rheometer (Anton Paar, MCR302, Austria) equipped with a parallel plate geometry (50 mm diameter, 0.5 mm gap) at 25 °C. Amplitude sweep tests were first conducted over a strain range of 0.01–100% at 1 Hz frequency to determine the linear viscoelastic region (LVR). All muffin batter formulations exhibited linear viscoelastic behavior between 0.1% and 10% strain; therefore, 0.1% strain was selected for frequency sweep tests. Frequency sweep tests (0.1–10 Hz) measured G′ (elastic modulus) and G″ (loss modulus) values, and the resulting data were fitted using non-linear regression based on the power law model, as described in Equations (6) and (7).

(6) $$G^{\prime }=K^{\prime }{\left(\omega \right)}^{n^{\prime }}$$

(7) $$G^{\prime \prime }=K^{\prime \prime }{\left(\omega \right)}^{n^{\prime \prime }}$$

where K′ and K″ are consistency indices (Pa s*^n^*), *n′* and n″ are the frequency exponents, and ω is the angular frequency (rad s^−1^). These parameters were used to compare the viscoelastic behavior between the different formulations [30]. Additionally, loss tangent (tan δ) was calculated as the ratio of G″ to G′ at an angular frequency of 1.36 rad s^−1^ to evaluate the viscoelastic balance of the samples. Flow behavior was evaluated via steady shear measurements conducted over a shear rate range of 0.01–100 s^−1^.

The crust and crumb color analysis of the muffin samples was performed using a Minolta Chroma Meter (CR-400, Osaka, Japan) based on the CIELab* color scale. In this method, the L* value represents lightness (where 0 = black and 100 = white), the a* value indicates redness–greenness, and the b* value corresponds to yellowness–blueness. For the internal color analysis, the muffins were vertically sliced through the center, and color measurements were taken from the central area of the cut surface. From these, Chroma (C*) and hue angle (h°) values were calculated to express color saturation and hue, respectively [31]. The total color difference (ΔE) between the reference (control) and treated samples was calculated using the following Equations:

(8) $$\Delta E=\sqrt{\left(L^{*}-L_{0}^{*})+{\left(a^{*}-a_{0}^{*}\right)}^{2}+(b^{*}-b_{0}^{*}\right)}$$

(9) $$C^{*}=\sqrt{\left(a^{*2}+L^{*2}\right)}$$

(10) $$h^{o}=arctan\left(\frac{b}{a}\right)$$

where L_0_*, a_0_*, and b_0_* represent the color values of the reference sample. The hue angle (h°) was interpreted based on the signs of a* and b*: 0° to 90°: a* > 0, b* > 0; 90° to 180°: a* < 0, b* > 0; 180° to 270°: a* < 0, b* < 0; 270° to 360°: a* > 0, b* < 0.

The microstructure of the muffin samples was examined using a field-emission scanning electron microscope (Thermo Scientific Apreo S, Thermo Fisher Scientific, Waltham, MA, USA). Muffin samples were freeze-dried and carefully fractured to expose internal crumb structures. Prior to imaging, the samples were mounted on aluminum stubs using carbon adhesive tape and sputter-coated with a thin layer of gold (~10 nm) using a Leica EM ACE600 coater (Leica Microsystems, Wetzlar, Germany) to ensure conductivity. SEM analysis was performed under high-vacuum mode at 10–15 kV acceleration voltage. Representative images were taken at 500× and 1000× magnifications for each sample.

#### 2.3.3. Chemical Composition

The moisture content (%) of the muffin samples was determined in accordance with AOAC [32]. For this analysis, 5 g of ground sample was uniformly distributed in pre-weighed aluminum drying dishes and subjected to drying at 105 ± 2 °C in a laboratory oven (Memmert UN110, Memmert GmbH + Co. KG, Schwabach, Germany) until a constant weight was reached. Moisture content was calculated by comparing the initial and final weights of the sample. The ash content (%) was determined following the standard procedure described in [33], in which the samples were incinerated at 550 °C using a muffle furnace (Nabertherm LV 9/11, Nabertherm GmbH, Lilienthal, Germany). Protein content (%) was measured using the Dumas combustion method with a Dumas Protein Analyzer (Velp NDA 701, Velp Scientifica Srl, Usmate Velate, Italy), in compliance with AOAC [34]. A nitrogen-to-protein conversion factor of 6.25 was used for the calculations. Total dietary fiber (%) was determined using the enzymatic–gravimetric method according to AOAC [35], employing a commercial assay kit (Megazyme, Bray, Ireland). The method involved sequential digestion with α-amylase, protease, and amyloglucosidase, followed by ethanol precipitation and gravimetric measurement of the residue. Total fat content (%) was determined according to the NMKL method No. 160 [36], which involves hydrolysis with hydrochloric acid, followed by fat extraction with petroleum ether and solvent evaporation. An automatic fat analyzer (Velp Ser148/6, Velp Scientifica Srl, Usmate Velate, Italy) was employed for this assessment. Carbohydrate content (%) was calculated using the following formula (Equation (11)) [37]:

(11) $$Carbohydrate\;\left(\%\right)=100-(Moisture+Ash+Protein+Fat+Dietary\;Fiber)$$

Energy content (kcal/100 g) was calculated according to the general Atwater system, as outlined by the Food and Agriculture Organization [37], using the following conversion factors: 4 kcal/g for protein, 9 kcal/g for fat, 4 kcal/g for available carbohydrate, and 2 kcal/g for dietary fiber (Equation (12)). The 2 kcal/g factor for dietary fiber is consistent with FAO guidance on the physiological energy contribution of fermentable fiber due to partial colonic fermentation. We acknowledge that some labeling standards treat dietary fiber as non-caloric; therefore, the energy values reported here reflect this methodological choice and could be modestly lower if fiber were assigned 0 kcal/g.

(12) Energy (kcal/100 g)=(Protein×4)+(Fat×9)+(Carbohydrate×4)+(Dietary Fiber×2)

All values are expressed on a wet weight basis.

The mineral content of Ca, K, Mg, Na, and P in the muffin samples was determined using an ICP-MS (Agilent 7500 CX, Agilent Technologies, Santa Clara, CA, USA). Samples (0.5 g) were digested in a microwave system (Ethos Up, Milestone Srl, Sorisole, Italy) using 65% HNO_3_ and 30% H_2_O_2_. A calibration curve with R^2^ > 0.999 was used in accordance with NMKL method No. 186 [38], and analyses were performed in triplicate for accuracy.

#### 2.3.4. Phenolic Extraction, In Vitro Digestion Model, Total Antioxidant Capacity (TAC), and Total Phenolic Content (TPC) Determination

Phenolic compounds in muffin samples were extracted by mixing 2 g of finely ground sample with 5 mL of 75% aqueous methanol, acidified with 0.1% formic acid. The mixture was shaken in a water bath (Memmert WNB 22, Memmert GmbH + Co. KG, Schwabach, Germany) at 20 °C for 15 min, then centrifuged (Sigma 3K30, Sigma Laborzentrifugen GmbH, Osterode am Harz, Germany) at 9000× *g* rpm for 10 min [39]. The extraction was repeated to maximize yield, and the extracts were stored at −20 °C for further analysis of total phenolic content (TPC) and total antioxidant capacity (TAC).

To evaluate bioaccessibility, an in vitro digestion model simulating digestion in the mouth, stomach, and small intestine was used [40]. In the oral phase, 10 g of the muffin sample was mixed with 10 mL of simulated salivary fluid (SSF), 0.2 mL of α-amylase solution (1500 U/mL), and 25 μL of 0.3 M CaCl_2_, then incubated at 37 °C for 2 min. In the gastric phase, the mixture was combined with 20 mL of simulated gastric fluid (SGF), 0.4 mL of pepsin solution (25,000 U/mL), and 5 μL of 0.3 M CaCl_2_, adjusted to pH 3.0 with 1 M HCl, and incubated at 37 °C for 2 h. In the intestinal phase, the gastric digest was mixed with 20 mL of simulated intestinal fluid (SIF), 1.0 mL of pancreatin–bile extract solution (containing 800 U/mL pancreatin and 10 mM bile salts), and 40 μL of 0.3 M CaCl_2_, adjusted to pH 7.0 with 1 M NaOH, and incubated at 37 °C for another 2 h. Aliquots were taken after each digestion phase and stored at −20 °C until TAC and TPC analyses.

TAC was measured using two spectrophotometric assays: DPPH and CUPRAC. In the DPPH assay, 0.1 mL of extract was added to 3.9 mL of freshly prepared 0.1 mM DPPH methanolic solution, vortexed, and incubated in the dark at room temperature (25 ± 2 °C) for 30 min. Absorbance was measured at 517 nm. In the CUPRAC assay, 1.0 mL of 10 mM CuCl_2_, 1.0 mL of 7.5 mM neocuproine (in ethanol), 1.0 mL of 1 M ammonium acetate buffer (pH 7.0), and 0.5 mL of sample extract were mixed and incubated for 30 min at room temperature (25 ± 2 °C). Absorbance was measured at 450 nm. Results are expressed as milligrams of Trolox equivalent (TE) per kilogram of dry matter. For quantification, calibration curves were constructed using Trolox standards in the ranges of 0–250 µmol/L for DPPH (R^2^ = 0.9998) and 0–1.0 mmol/L for CUPRAC (R^2^ = 0.9997).

The total phenolic content (TPC) was determined using the Folin–Ciocalteu method. Briefly, 0.5 mL of extract was mixed with 2.5 mL of 10% Folin–Ciocalteu reagent and incubated for 5 min at room temperature. Then, 2.0 mL of 7.5% (*w*/*v*) sodium carbonate solution was added, and the mixture was incubated in the dark at room temperature (25 ± 2 °C) for 60 min. Absorbance was measured at 765 nm. A calibration curve was prepared using gallic acid standards ranging from 0 to 250 mg/L (R^2^ = 0.9947). TPC results were expressed as milligrams of gallic acid equivalent (GAE) per 100 g of sample [41,42,43].

The bioaccessibility of phenolic compounds was calculated as the ratio of TPC and TAC values of the digested samples to those of the undigested samples, and expressed as a percentage.

#### 2.3.5. Amino Acid (AA) Composition

The amino acid composition was determined following the protocol described in the Agilent Amino Acid Analysis Application Guide [44] and supported by the approach reported in Drabińska [45], with slight modifications as detailed below. The amino acid composition of the muffin samples was determined using an Agilent 1260 Infinity II high-performance liquid chromatography (HPLC) system equipped with a diode array detector (DAD). Approximately 0.5 g of muffin samples were homogenized in an IKA Ultra-Turrax (T25, Staufen im Breisgau, Germany) with 25 mL of 0.1 N HCl in an ice bath with dry ice for 8 min. The homogenates were centrifuged at 5240× *g* for 20 min at 4 °C, and the supernatants were filtered through 0.45 μm PVDF membranes. For protein precipitation, 200 μL of the extract was mixed with 800 μL of acetonitrile and centrifuged again under the same conditions. The filtrates were collected into amber vials for analysis.

For total amino acid determination, 0.5 g of sample was also subjected to acid hydrolysis with 5 mL of 6 N HCl containing 250 μL of 2 mM phenol (to prevent oxidation) and 2 mL of 2% (*w*/*v*) 2,2′-dithiobis(5-nitropyridine) (DTDPA, to stabilize sulfur-containing amino acids such as cystine and methionine). The mixture was hydrolyzed in sealed tubes at 110 °C for 24 h. Following hydrolysis, the solution was neutralized to pH 6.7–7.3, diluted to 100 mL with distilled water, centrifuged at 4000× *g* rpm for 5 min, and filtered if necessary. One milliliter of the resulting solution was transferred into vials for HPLC analysis [44].

Prior to injection, amino acids were derivatized using pre-column reagents including o-phthalaldehyde (OPA), fluorenylmethyloxycarbonyl chloride (FMOC), and 3-mercaptopropionic acid (3-MPA). Separation was performed on an Agilent ZORBAX Eclipse AAA column (4.6 × 150 mm, 5 µm) at 40 °C with a flow rate of 1.5 mL/min. The mobile phases consisted of sodium phosphate buffer (pH 7.8) and a mixture of acetonitrile/methanol/water (45:45:10, *v*/*v*/*v*). The injection volume was 10 µL. Peaks were detected at 338 nm using OpenLAB CDS ChemStation software (version: C.01.07, Agilent Technologies, Santa Clara, CA, USA).

Quantification was performed by comparing sample peak areas with those of a certified amino acid standard mixture (Agilent, USA) containing 17 amino acids at a concentration of 2.5 mM each. Calibration curves were prepared for each amino acid over the range of 0.1–2.5 mM with R^2^ values exceeding 0.9990. Standards were stored at −20 °C and protected from light [44].

The results were expressed as g amino acid per 100 g sample. Both essential and non-essential amino acids were quantified. Tryptophan was also detected in the hydrolysate, indicating total amino acid composition.

#### 2.3.6. Shelf-Life Analysis

To assess real-time shelf stability, the baked muffins were first cooled and then stored in sealed, double-layer polyethylene bags under ambient conditions (25 ± 2 °C, 60 ± 5% relative humidity) for 30 days. Shelf life evaluations were conducted at three time points (days 0, 15, and 30), as these intervals are commonly used to monitor the quality deterioration of bakery products during short-term storage [7]. All physical and chemical analyses were performed as described in Section 2.3.2 and Section 2.3.3. Moisture content, ash, total fat, texture, color, and microbiological parameters were assessed on days 0, 15, and 30.

For microbial testing, one gram of each sample was homogenized in peptone water, serially diluted up to 10^−2^, and plated using the spread plate technique. Analyses included total mesophilic aerobic microorganisms, *Escherichia coli*, *Salmonella* spp., *Staphylococcus aureus*, and molds and yeasts. Total mesophilic counts were performed on Plate Count Agar (PCA) at 30 °C for 72 h. Molds and yeasts were enumerated on Dichloran Rose Bengal Chloramphenicol Agar (DRBCA) at 25 °C for five days. Detection of *E. coli* and *Salmonella* spp. involved selective enrichment and plating on Tryptone Bile Glucuronide Agar (TBX), with incubation at 44 °C for 18–24 h and 37 °C for 24 h, respectively. *Staphylococcus aureus* was isolated on Baird-Parker Agar (BPA) at 37 °C for 48 h and confirmed using a coagulase test.

All microbiological analyses were conducted under aseptic conditions to prevent contamination. Pathogenic microorganisms were evaluated according to typical regulatory limits: *Salmonella* spp. and *E. coli* were considered negative when absent in 25 g of sample, and *S. aureus* levels below 10 CFU/g were considered acceptable.

#### 2.3.7. Sensory Evaluation of Muffins

A trained panel consisting of 24 individuals (12 females and 12 males) aged 18–47 participated in the sensory evaluation. All panelists were students or academicians from the Department of Gastronomy and Culinary Arts at Maltepe University, regular consumers of muffin products, and trained according to international sensory evaluation standards (ISO 3972; ISO 8586; ISO 11132) [46,47,48]. Muffin samples were placed on plastic plates and coded with randomly assigned three-digit numbers. Evaluations were conducted based on seven sensory attributes: crust color, crumb (inner) color, aroma, taste/flavor, appearance, softness, and overall acceptability. A 9-point hedonic scale was used, where 1 represented “extremely poor” and 9 represented “excellent.” Samples with an average score of 5 or higher were considered acceptable and suitable for consumption. All evaluations were carried out in individual sensory booths under controlled environmental conditions (22 ± 1 °C, neutral lighting). Unsalted breadcrumbs and water at room temperature were provided to cleanse the palate between samples. Ethical approval for the sensory analysis was obtained from Maltepe University on 28 November 2024 (Approval No: 2024/22−09).

#### 2.3.8. Statistical Analysis

All measurements were conducted in triplicate (n = 3) unless otherwise stated. Sensory evaluation was conducted with 24 trained panelists, and each sample was evaluated once per panelist under controlled conditions. The formulations and experimental design details are provided in Table 1. Results were expressed as mean values ± standard deviations. Statistical analyses were conducted using SPSS software (version 16.0; SPSS Inc., Chicago, IL, USA). To assess the impact of different plant-based egg substitutes on the measured parameters, ANOVA was employed. Tukey’s post hoc test was applied to determine significant differences among group means at a significance level of *p* < 0.05.

To investigate the interrelationships among physicochemical, structural, nutritional, and bioactive properties, Pearson’s correlation coefficients were calculated. Multivariate data exploration was conducted using principal component analysis (PCA) to identify patterns and visualize clustering behavior among the formulations. Hierarchical cluster analysis (HCA) was employed using Ward’s linkage method and Euclidean distance as the similarity metric to group samples based on their overall functional characteristics. Both PCA and HCA were initially conducted using SPSS software, and corresponding dendrograms and visualizations were refined in OriginPro 2025 for enhanced graphical presentation.

## 3. Results and Discussion

### 3.1. Rheological Properties of Muffin Batters

**Figure 1:**
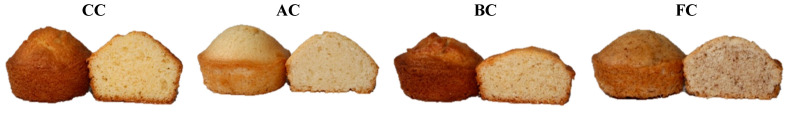

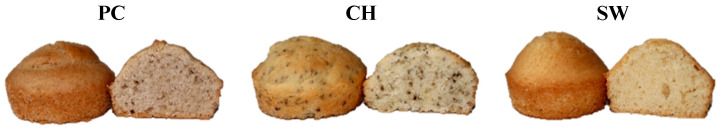
Muffin samples formulated with egg and plant-based substitutes. CC: Control muffin, AC: Aquafaba muffin, BC: Banana muffin, FC: Flaxseed muffin, PC: Psyllium muffin, SWC: Soapwort muffin, CHC: Chia muffin.

The rheological properties of the muffin batters—including fluidity, elasticity, and viscosity—were analyzed using a rheometer to evaluate the impact of different egg substitutes (Figure 1).

Inappropriate batter viscosity can negatively affect muffin quality. High viscosity limits gas expansion, while low viscosity leads to structural collapse. A well-balanced batter ensures proper aeration and a stable texture during baking. The consistency of the muffin batter was examined using steady-shear testing [49].

**Figure 2:**
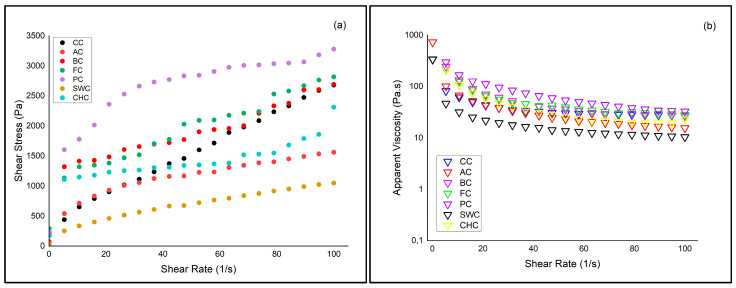
Flow behavior of vegan muffin samples: (**a**) Shear stress vs. shear rate, (**b**) apparent viscosity vs. shear rate. CC: Control muffin, AC: Aquafaba muffin, BC: Banana muffin, FC: Flaxseed muffin, PC: Psyllium muffin, SWC: Soapwort muffin, CHC: Chia muffin.

The relationship between shear stress and apparent viscosity with varying shear rate is illustrated in Figure 2. All muffin batter samples showed shear-thinning (pseudoplastic) behavior, with decreasing viscosity and increasing shear stress as shear rate increased. The PC (psyllium) and FC (flaxseed) batters displayed relatively higher shear stress and viscosity values compared to others. This suggests that these ingredients contribute to a more cohesive internal structure, primarily because of their strong water-binding and gel-forming properties. The incorporation of psyllium gum into gluten-free batter formulations has been shown to significantly increase batter viscosity and improve moisture retention, softness, and volume of muffins, supporting its role in strengthening batter structure [14]. Similarly, flaxseed gel has demonstrated the ability to replace eggs in muffin formulations without compromising texture or moisture. In a previous study, muffins prepared with up to 50% flaxseed gel retained comparable cohesiveness and structural properties to egg-based controls, supporting its potential as an effective egg replacer [50].

In contrast, SWC (soapwort) and AC (aquafaba) had much lower values, suggesting weaker network formation and reduced resistance to deformation. This may be attributed to the limited network-forming ability of these ingredients. Although soapwort contains saponins that contribute to foam formation, its inclusion in muffin batter formulations has not shown significant improvements in rheological or textural properties. It is reported that even with up to 75% replacement of egg white by soapwort extract, there were no notable changes in batter consistency or muffin firmness, indicating a limited role in network strengthening [23]. Likewise, while aquafaba offers emulsifying and foaming functions, studies have demonstrated that muffins made with aquafaba have lower springiness, chewiness, and volume compared to egg-based controls, reflecting reduced structural integrity [25,51].

These findings confirm that egg replacers vary significantly in their ability to support the viscoelastic structure of muffin batters. Ingredients rich in hydrocolloids were more effective in maintaining flow behavior and batter consistency. These properties are essential for achieving proper muffin volume and uniform crumb structure. The results demonstrate that rheological performance is highly dependent on the type of plant-based ingredient and its interaction with starch, proteins, and fibers within the batter matrix [50,52].

**Figure 3:**
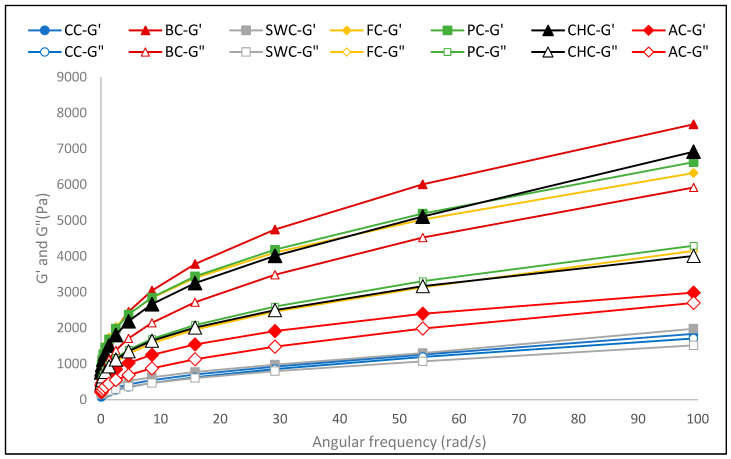
The rheological parameters of the batter sample, elastic modulus (G′), and viscous modulus (G″). CC: Control muffin, AC: Aquafaba muffin, BC: Banana muffin, FC: Flaxseed muffin, PC: Psyllium muffin, SWC: Soapwort muffin, CHC: Chia muffin.

Beyond steady-shear behavior, dynamic oscillatory measurements were also conducted to further evaluate the viscoelastic properties of the batters. Storage modulus (G′) and loss modulus (G″) were frequency-dependent, showing shear-thinning behavior and structural degradation at higher frequencies (Table 2 and Figure 3). The G′ value represents the elastic behavior of a sample, while the G″ value indicates its viscous behavior [53]. The CC and SWC samples had the lowest G′ and G″ values, suggesting a more fluid batter, while the BC sample had the highest G′ value, indicating stronger elasticity and a solid-like structure. However, despite its saponin-rich composition that primarily contributes to foaming, SWC was less effective in strengthening viscoelastic behavior compared to hydrocolloid and mucilage-rich replacers such as psyllium or flaxseed, which provide stronger water-binding and gel-forming capacity [23]. Consequently, muffin batters containing different egg substitutes may better retain structure after deformation. As shown in Table 2, the BC sample exhibited the highest storage (G′ = 305.13 Pa) and loss (G″ = 217.01 Pa) moduli, indicating a well-developed viscoelastic network. In contrast, the CC sample had the lowest values (G′ = 66.11 Pa; G″ = 48.44 Pa). Among the plant-based formulations, the CHC sample showed the strongest viscoelastic response (G′ = 262.26 Pa; G″ = 187.11 Pa), followed by PC, FC, AC, and SWC.

Previous studies have shown that plant-based proteins can enhance batter firmness by increasing both G′ and G″ values [16,54]. Similarly, muffins formulated with egg substitutes exhibited higher elasticity and viscosity compared to conventional formulations. The incorporation of plant-based proteins has been particularly associated with improved viscoelastic properties due to their ability to form structured gel networks [55]. In this study, the BC batter demonstrated the strongest network structure, while batters containing chia, psyllium, and flaxseed gels also showed notable enhancements in viscoelastic behavior. The choice of egg substitute significantly affected the rheological performance of the batter, influencing its elasticity, viscosity, and textural characteristics.

Loss tangent (tan δ) values, which represent the ratio of viscous to elastic behavior (G″/G′), provide insights into the viscoelastic balance of the muffin batters. Lower tan δ values indicate more elastic (solid-like) structures, whereas higher values suggest more viscous (fluid-like) behavior [56]. The loss tangent of vegan batter formulations ranged from 0.551 to 0.845. As all batters showed, the loss tangent was lower than 1. Among the samples, the FC batter had the lowest tan δ (0.551), indicating a predominantly elastic behavior and a well-developed gel-like network. Similarly, CHC and PC samples also had relatively low tan δ values (0.621 and 0.598, respectively). In contrast, the CC sample had the highest tan δ value (0.845), and SWC also had a higher tan δ (0.748). Notably, BC and AC displayed moderate tan δ values (0.707 and 0.706). Studies reported that both plant-based (banana, chia seed, soymilk powder) and egg-containing muffin batters had low tan δ values (<1), indicating predominantly elastic behavior. Unlike that study, in the present work, the control batter with egg had the highest tan δ value, while several plant-based formulations demonstrated more elastic characteristics, suggesting that viscoelastic responses can vary depending on formulation context and the nature of the egg replacer [17,54]. This contrast highlights that the impact of egg replacers on batter rheology is not uniform and may depend on interactions with other ingredients, such as starch type, fiber content, or protein functionality. It also suggests that certain plant-based alternatives—such as flaxseed, psyllium, or chia—may contribute to forming cohesive, elastic structures comparable to or even surpassing those formed by egg in specific formulations.

### 3.2. Physical Properties of Muffin Samples

The physical properties and appearance of the muffins are presented in Table 3 and Figure 1, respectively. After baking, the lowest weight loss was observed in the BC sample (4.83%), while the highest was observed in the SWC sample (9.23%). The CC, AC, and BC samples exhibited lower moisture loss, whereas the FC, PC, SWC, and CHC samples had higher values. The FC and PC samples, containing flaxseed and psyllium, respectively, exhibited higher moisture loss. This may be attributed to the high soluble fiber content of these ingredients, which can retain water in the batter but may release it during baking due to their swelling and gelling properties [17]. In contrast, the SWC and AC samples showed relatively lower moisture loss, which may be linked to the foaming ability of *Saponaria officinalis* and aquafaba, respectively, as both ingredients are known to stabilize air and moisture within the batter matrix [15,16].

The volume index (VI), representing the average muffin height, was highest in the CC sample (119.00 mm), followed by the SWC (108.00 mm) and AC (100.67 mm) samples, indicating satisfactory leavening. The CHC (84.00 mm) and BC (88.33 mm) samples had the lowest VI values, which may reflect reduced expansion. While no official threshold exists for VI, values above 100 mm are often associated with favorable muffin structure, particularly in egg-containing formulations where protein-based foaming and coagulation mechanisms enhance volume. Studies by Agrahar-Murugkar et al. [17] and Rahmati and Mazaheri Tehrani [57] similarly reported that cakes formulated with sprouted flours, banana, or soy milk had lower VI values compared to those containing eggs, likely due to increased batter density and reduced incorporation of air during mixing. Statistical analysis confirmed that the VI values differed significantly among samples (*p* < 0.05), supporting the visual distinctions in muffin height (Figure 1).

The symmetry index (SI), calculated as the absolute difference between the left and right edge heights, ranged from 4.67 to 10.33 mm. No negative SI values were recorded, suggesting that no sample experienced structural collapse during baking [58,59]. As reported by Dadalı and Elmacı [60], positive SI values are typical in cakes, indicating a higher center relative to the edges, which is a result of expansion and structural setting during baking. However, excessive SI values may reflect surface asymmetry caused by uneven batter distribution or heat gradients. In this study, the BC sample, with an SI of 4.67 mm, exhibited the most symmetrical profile, while the AC, FC, and SWC samples had higher SI values (≥10 mm), pointing to more curved or uneven surfaces. These differences were also statistically significant (*p* < 0.05), indicating that the type of egg substitute had a measurable impact on surface symmetry.

The uniformity index (UI), calculated as the mean absolute difference between the center and edge heights, is a measure of lateral symmetry and surface evenness of muffins. Ideally, UI should be as close to zero as possible, indicating uniform rise and balanced surface formation. In this study, UI values ranged from 0.00 to 1.00 mm, with BC, FC, and SWC samples having values of 0.00 mm. This result indicates a highly symmetrical structure, where the center and edges were nearly identical in height. These findings align with previous work showing that such low UI values correspond to uniform muffin morphology [60]. Furthermore, statistical analysis revealed no significant differences among the groups (*p* > 0.05), supporting the conclusion that the type of egg substitute did not markedly affect muffin uniformity.

According to the rheological analysis, samples with higher G′ values tended to form firmer batters, which limited air retention and ultimately reduced the final muffin volume. The BC sample, which had the highest G′ value, also had low VI and SI values, supporting the idea that firmer batters restrict bubble incorporation and expansion during baking [61]. This observation aligns with its moderate tan δ value (0.707), suggesting that elastic behavior is still dominant, though less pronounced than in other plant-based batters. The viscoelastic balance observed in this sample likely limited air cell expansion during baking, contributing to its reduced muffin volume.

This study evaluated the effects of various egg substitutes on the textural properties of muffins, with the results presented in Table 3. The findings showed that egg substitutes significantly influenced parameters such as hardness, springiness, cohesiveness, and chewiness. The highest hardness value was recorded in the CHC sample (2735.73 gf), whereas the lowest was observed in SWC (1266.15 gf). Most egg substitutes increased hardness, likely due to differences in water retention capacities, particularly related to their fiber content [62]. Springiness measurements revealed that the elasticity typically provided by eggs could not be fully replicated by plant-based alternatives. Notably, BC and CHC samples exhibited reduced springiness. The highest cohesiveness value was found in the CC sample (0.70), while the BC sample had the lowest (0.50). These findings suggest that plant-based ingredients interact differently with the muffin matrix, affecting binding behavior and internal structure [12]. Regarding chewiness, CHC had the highest value (1012.27), whereas SWC had the lowest (529.96). In conclusion, the results indicate that plant-based egg substitutes substantially alter the textural characteristics of muffins, highlighting the importance of optimizing formulations based on the desired texture profile.

Color is a critical quality attribute in muffins, influenced by both Maillard and caramelization reactions. While crust color is largely determined by sugar–amino acid interactions, the internal color is shaped by the composition of ingredients [25,63]. In this study, the CIELAB color system was used to evaluate crust and crumb color, with the results summarized in Table 3. Crust brightness (L*) ranged from 56.17 to 73.17, with the SWC sample having the highest value (73.17), likely due to the whitening effect of soapwort extract [23]. The AC sample also exhibited increased crust brightness compared to the control (CC), aligning with previous findings that chickpea aquafaba enhances lightness [21]. Despite uniform baking conditions, the SWC sample retained more moisture, which may have contributed to its higher L* value. Across all samples, the crumb appeared more reddish and yellowish than the crust. Egg substitutes led to a decrease in crust a* and b* values, with the CC sample having the highest a* (12.47) and b* (30.99) values. Since the internal muffin temperature does not exceed 100 °C, Maillard reactions are minimal, and observed color differences are primarily attributed to raw ingredients [59]. The yellowish tone of the CC sample can be attributed to the carotenoids present in egg yolk [54]. Among the samples, AC, BC, and SWC had similar internal b* values (*p* > 0.05), and appeared most yellowish after CC. These results are consistent with previous studies showing that muffins made with flaxseed and psyllium produce statistically comparable crumb color values [64].

In addition to the L*, a*, and b* values, Chroma (C*), hue angle (h°), and total color difference (ΔE) were calculated to better capture visual differences. The control muffin (CC) exhibited the most vivid color in both crust (C* = 33.87) and crumb (C* = 24.39), while PC and FC samples had significantly lower C* values, reflecting weaker tones. Hue angle analysis showed that crust colors shifted from orange–yellow (CC: 68.41°) to more yellowish hues in FC (88.76°) and CHC (85.57°). For the crumb, CC and AC had hue values around 270°, which mathematically correspond to blue–green hues in the CIELAB space. However, these high hue values primarily result from low negative a* values combined with dominant positive b* values. In the crust, the greatest ΔE differences were observed in CHC (23.55) and FC (23.42). In the crumb, ΔE remained perceptible in PC (20.70) and SWC (17.49), indicating that the use of egg substitutes in muffin formulations leads to visually noticeable color changes. Notably, the crust ΔE for BC was lower than for the other replacers (9.44). This can be attributed to the natural sugar content of banana puree, which promotes Maillard and caramelization pathways in a direction similar to the CC, while avoiding the hue/chroma shifts induced by hydrogel-based replacers (psyllium, flaxseed, chia). As a result, the color vector remained closer to that of the control.

### 3.3. Morphology

**Figure 4:**
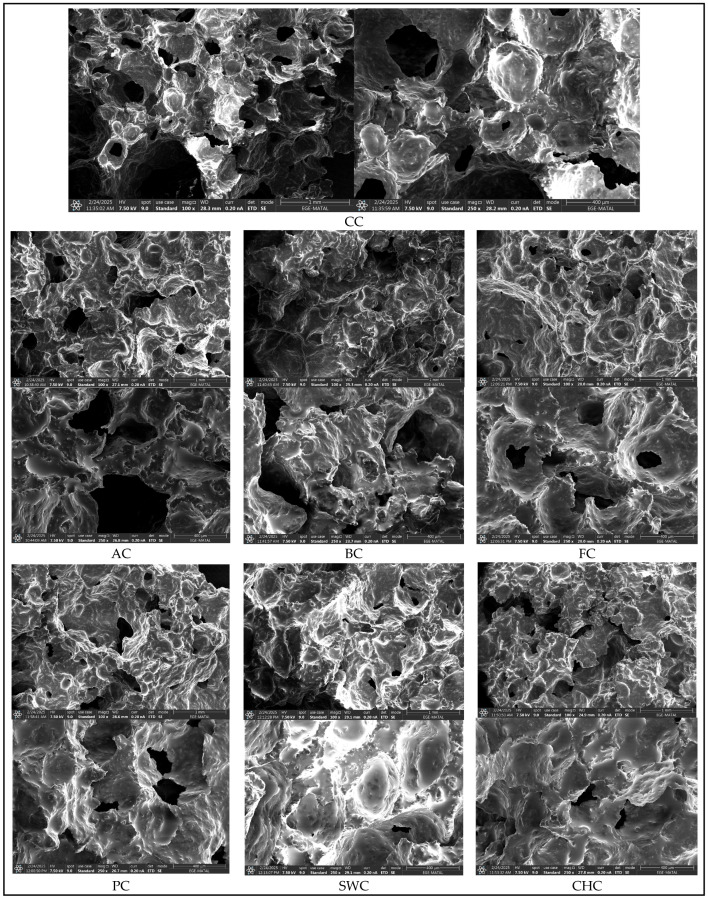
SEM micrographs of muffin samples captured using a scanning electron microscope (SEM) with magnifications of ×100 and ×250. CC: Control muffin, AC: Aquafaba muffin, BC: Banana muffin, FC: Flaxseed muffin, PC: Psyllium muffin, SWC: Soapwort muffin, CHC: Chia muffin.

In this study, the effects of different egg substitutes on the microstructure of muffins were analyzed using scanning electron microscopy (SEM) (Figure 4).

The CC and AC samples exhibited a well-organized protein network with a homogeneous pore structure, while the BC and CHC samples showed larger and more irregular pores, likely due to their starch-dominant compositions. Furthermore, notable differences in protein–carbohydrate interactions were observed among the PC, FC, and SWC samples. The PC sample formed a dense and compact network; in contrast, FC exhibited an irregular pore structure, and SWC produced larger and more interconnected pores, possibly related to its high air retention capacity. The CC and AC samples demonstrated the most stable protein matrix, whereas polysaccharide-rich alternatives such as flaxseed (FC), psyllium (PC), and soapwort (SWC) resulted in marked alterations in pore morphology.

These findings highlight the need for further comprehensive analysis to better understand how alternative ingredients affect muffin microstructure. Supporting this conclusion, previous SEM studies on pound muffins reported that most commercial egg replacers are starch-based, while protein-rich alternatives tend to form globular structures [65]. Ultimately, the structural differences among egg substitutes significantly influence the physical and textural properties of muffins.

### 3.4. Chemical Composition of Muffin Samples

The chemical composition of the muffin samples varied significantly in terms of moisture, ash, protein, fat, dietary fiber, carbohydrate, and energy content, depending on the type of egg substitute used (Table 4).

Moisture content ranged from 13.58 to 19.06 g/100 g, with the SWC sample having the highest value and AC the lowest. All samples, except AC, had higher moisture content than the control (CC). These variations in moisture content may be influenced by the water-holding capacity of the ingredients used. Ash content was significantly higher in all samples compared to the control (CC), with CHC having the highest value (*p* < 0.05), likely due to the elevated levels of organic and inorganic compounds in chia seeds. Protein content ranged from 5.24 to 8.54 g/100 g and was lower in all samples than in CC, which is consistent with the plant-based nature of the egg substitutes. This observation aligns with previous findings on legume-based aquafaba and chia seed formulations [16,66]. Nevertheless, incorporating protein fortification strategies may help improve the nutritional profile of such formulations. Fat content ranged from 18.78 to 22.16 g/100 g and was generally lower than that of CC. No statistically significant differences were observed among AC, BC, PC, and CHC (*p* > 0.05), supporting the potential of these alternatives in developing reduced-fat bakery products. In addition, it should be noted that the control (CC) was prepared with cow’s milk, whereas almond milk was used in the plant-based formulations. Since cow’s milk and eggs are naturally richer in protein and fat, their replacement with plant-based substitutes and almond milk further explains the observed reductions.

Total dietary fiber (TDF) content was highest in the FC and PC samples (1.50 g/100 g), significantly exceeding that of the control (CC; 0.73 g/100 g), which can be attributed to the fiber-rich properties of flaxseed and psyllium [19,67]. Energy values ranged from 414.87 to 448.53 kcal, with the CC sample having the highest and SWC the lowest (representing a 7.50% reduction), primarily due to lower fat content. Although the energy content of all other samples was also lower than that of CC, the differences were not statistically significant (*p* > 0.05).

Macroelements such as sodium (Na), potassium (K), calcium (Ca), magnesium (Mg), and phosphorus (P) are required by the body in relatively large amounts to maintain basic physiological functions. Calcium and magnesium are important for the structure of bones and teeth, and also take part in carbohydrate and protein metabolism. Phosphorus, the second most abundant mineral in bones after calcium, contributes to energy metabolism, nerve transmission, and acid–base regulation. Potassium is the main intracellular cation and plays a role in muscle contraction, pH balance, and ionic regulation. Sodium, as the major extracellular cation, helps control osmotic pressure and supports the transport of water and electrolytes [68]. The mineral composition of the muffin samples varied significantly depending on the egg substitutes used. The P, Na, Mg, K, and Ca values of the muffin samples are presented in Table 4. The highest phosphorus (2188.36 mg/100 g) and sodium (2001.69 mg/100 g) contents were found in the CC sample, while SWC had the lowest values. The use of plant-based egg alternatives significantly reduced sodium content (*p* < 0.05). Magnesium content ranged from 98.99 to 208.73 mg/100 g, with CHC having the highest level, consistent with the high magnesium content of chia seeds [69]. Potassium content was highest in the BC sample (1352.42 mg/100 g) and lowest in SWC, while calcium content was highest in CHC (372.00 mg/100 g). Notably, the AC, FC, PC, and CHC formulations generally led to elevated levels of magnesium (Mg), potassium (K), and calcium (Ca), while sodium (Na) content tended to decrease. This pattern reflects the natural mineral composition of the plant-based ingredients used in these formulations. For example, chia seeds and flaxseed are naturally rich in calcium and magnesium, and also contain a high potassium-to-sodium ratio, which helps explain the increased mineral content in the respective muffin samples [54,66]. Banana, commonly used as an egg substitute, is known for its high potassium content (358/100 g), which may have contributed to the enhanced K levels observed in the banana-based formulation [70]. These ingredients not only support improved mineral intake but may also offer additional nutritional benefits in plant-based baked products.

### 3.5. Total Phenolic Content and Antioxidant Capacity and In-Vitro Bioaccessibility

In the undigested state, the highest total phenolic content (TPC) was measured in the AC and CC samples, followed by BC and SWC (Table 5). The relatively high TPC in BC may be attributed to the presence of phenolic compounds such as catechins, gallic acid, and chlorogenic acid, which are naturally found in banana [71]. Similarly, soapwort, used in SWC, has been reported to contain saponins and various flavonoids and exhibit notable antioxidant potential [72]. Similarly, among the undigested samples, BC exhibited the highest antioxidant activity by DPPH (85.599 µmol TE/g), whereas CUPRAC values were relatively high in AC (0.137 µmol TE/g), BC (0.110 µmol TE/g), and PC (0.148 µmol TE/g). These results suggest that different egg substitutes significantly influence the antioxidant profile of the muffins, depending on the specific antioxidant mechanism involved.

Additionally, clear differences were observed between DPPH and CUPRAC assays. While DPPH primarily detects the radical-scavenging capacity of small, hydrophobic molecules in an organic medium, the CUPRAC method evaluates the total reducing power, including thiol-type antioxidants and hydrophilic compounds. Moreover, the CUPRAC reagent does not react with simple sugars due to its lower electrode potential, making it more selective for phenolic-based antioxidants [73]. This methodological distinction likely explains why samples such as AC had disproportionately higher CUPRAC values despite relatively low DPPH activity. Given their different sensitivities and mechanisms, combining assays such as DPPH and CUPRAC offers a more comprehensive assessment of antioxidant potential, especially in digested food systems [74].

The bioaccessibility of phenolics exhibited notable differences following in vitro digestion. The highest increase in TPC after intestinal digestion was observed in CHC (403.226%) and PC (362.069%), indicating enhanced release or transformation of bound phenolics. Although FC showed the highest TPC after the gastric phase, a subsequent reduction was noted in the intestinal phase, which may result from degradation of certain polyphenols under alkaline conditions [40,75]. While TPC increased significantly in CHC and PC and moderately in CC (*p* < 0.05), no statistically significant changes were observed in AC, SWC, or BC (*p* > 0.05). This outcome may be attributed to the limited release of bound phenolics during digestion, as previously reported for fiber-dense food matrices [75].

After intestinal digestion, CUPRAC-based antioxidant capacity was notably high in AC (1.016 µmol TE/g), CC (0.633 µmol TE/g), and SWC (0.601 µmol TE/g). In particular, AC exhibited a marked increase compared to its undigested value (0.137 µmol TE/g), representing approximately a 7.4-fold enhancement. Similarly, CHC and SWC also showed measurable improvements, with CUPRAC values increasing from 0.076 to 0.557 µmol TE/g and from 0.071 to 0.601 µmol TE/g, respectively. In contrast, FC and PC showed reduced antioxidant capacity after the gastric phase and had only moderate CUPRAC values post-intestinal digestion (0.363 and 0.387 µmol TE/g, respectively). This reduction may be attributed to degradation of sensitive antioxidant compounds under acidic or alkaline pH, or due to limited enzymatic breakdown of matrix-bound forms during digestion [76].

CUPRAC values were consistently higher than DPPH values across all digested samples, likely due to the assay’s ability to detect a broader spectrum of antioxidant species formed during enzymatic breakdown and pH shifts in the gastrointestinal tract [77]. These differences underscore the value of combining complementary antioxidant assays to fully capture the complex impact of in vitro digestion on antioxidant capacity.

In contrast to the CUPRAC assay, DPPH values declined markedly following in vitro digestion in nearly all samples. For instance, the DPPH activity of BC dropped from 85.60 to 1.13 µmol TE/g, corresponding to a 98.7% reduction. Similarly, AC showed a 97.6% decrease from 50.89 to 1.19 µmol TE/g, while FC declined by 98.0%. The observed reductions are likely linked to the degradation of low-molecular-weight antioxidants, such as catechins and chlorogenic acid, under the harsh pH conditions of digestion. A recent study on green bananas demonstrated a significant reduction in DPPH radical scavenging activity during in vitro digestion, which was attributed to the degradation of phenolic compounds such as chlorogenic acid under varying pH conditions [78]. A similar decrease in DPPH activity post-digestion was observed in chia seeds [79]. In our samples, CHC and PC retained relatively higher DPPH activity post-digestion (1.83 and 1.82 µmol TE/g, respectively), suggesting better stability of their antioxidant compounds or more effective release from the food matrix. This pattern highlights that the digestive environment, especially the pH change from gastric to intestinal phases, plays a key role in the stability of phenolic antioxidants [80].

These results highlight the dynamic changes in antioxidant behavior during digestion. CHC and PC showed high TPC bioaccessibility (403% and 362%, respectively), while SWC and AC retained strong CUPRAC activity (846% and 742%, respectively). Similar trends have been reported for chia and psyllium, which enhance antioxidant capacity through their high fiber content and strong binding properties [13,20]. In contrast, antioxidant losses in aquafaba-based samples may result from polyphenol–protein interactions that limit phenolic bioavailability [81].

### 3.6. Total Amino Acid (AA) Composition

Analysis of the total amino acid profile of the muffin samples, as presented in Table 6, revealed notable differences between the control (CC) and the vegan formulations. Amino acids are the building blocks of proteins. They can be grouped into neutral (e.g., alanine, valine, leucine), acidic (e.g., aspartic acid, glutamic acid), and basic types (e.g., arginine, lysine, histidine) according to the nature of their side chains. In addition to forming protein structures, they participate in various physiological functions such as energy metabolism, immune response, neurotransmission, and tissue repair [82].

The results indicate that the CC sample, which contains eggs, had significantly higher levels of certain essential amino acids compared to the vegan formulations. For example, the leucine content in CC was 0.330 g/100 g, whereas it was 56.7% lower in CHC (0.143 g/100 g) and 18.8% lower in BC (0.270 g/100 g). Similarly, lysine content in CC (0.114 g/100 g) was significantly higher than in CHC (0.008 g/100 g), corresponding to a 93% reduction. These differences are consistent with the inclusion of eggs, a complete protein source, rich in essential amino acids [83].

In contrast, the vegan muffin samples exhibited greater variability in amino acid composition, with some samples showing elevated levels of specific amino acids. For instance, FC had the highest arginine content (1.455 g/100 g), which was 44.5% higher than CC (1.007 g/100 g). Arginine is a non-essential amino acid with important physiological roles in nitric oxide synthesis and immune regulation [84]. In addition, the FC and SWC samples contained the highest glutamic acid levels (0.926 g/100 g and 0.924 g/100 g, respectively), nearly doubling the value found in CC (0.476 g/100 g). This amino acid plays a central role in neurotransmission and metabolic processes [85].

Despite these advantages, the amino acid profiles of vegan muffins also revealed reduced levels of certain essential amino acids in specific samples. For example, methionine was below the limit of detection in CHC, and significantly lower in AC (0.053 g/100 g) and BC (0.062 g/100 g) compared to CC (0.088 g/100 g), showing decreases of 39.7% and 29.5%, respectively. This is a notable observation, as methionine is critical for protein synthesis and acts as a precursor for cysteine and other sulfur-containing compounds [86].

Another distinctive feature was the presence of hydroxyproline in the AC (0.065 g/100 g), CHC (0.035 g/100 g), and PC (0.083 g/100 g) samples, while it was undetectable in CC. Hydroxyproline is typically abundant in collagen, and its presence may indicate the use of plant-based collagen analogues or hydrolyzed protein ingredients, which could contribute to the structural properties of these muffins [87].

It is important to emphasize that only the egg component was substituted in these formulations, while all other ingredients, including milk, remained constant. Therefore, the observed variations in amino acid profiles are primarily attributable to the nature of the egg replacers. This finding is consistent with the well-established fact that egg protein is considered a complete protein source, as it contains all essential amino acids in balanced and sufficient amounts for human nutrition. Plant-based proteins used in these substitutes may vary in amino acid completeness and balance, often lacking sufficient levels of one or more essential amino acids when compared to animal-based sources such as egg protein. The decision to determine amino acid compositions using baked muffins rather than raw ingredients was made to most accurately reflect the nutritional value of the product in its final consumable form. Baking processes, including Maillard reactions, amino acid–sugar interactions, and heat-induced degradation, can alter amino acid levels, while chemical interactions among ingredients (e.g., protein–sugar or protein–lipid interactions) may further influence these profiles. Therefore, analyzing the final product provides a realistic representation of the nutritional value that reaches the consumer, allowing a more accurate evaluation of the effects of different egg replacers on protein composition and amino acid balance.

### 3.7. Shelf-Life Evaluation of Muffin Samples

Throughout the 30-day storage period, notable differences emerged among the muffin formulations in terms of physicochemical, textural, color, and microbiological attributes (Supplementary Tables S1 and S2). Moisture content generally declined over time across all samples. The most obvious reduction was observed in the SWC formulation (from 19.06% to 9.22%), whereas the smallest decrease occurred in the AC sample (from 13.58% to 12.16%). This suggests that the SWC exhibited a higher susceptibility to moisture loss, while AC maintained a more stable hydration profile. This difference may be attributed to the compositional properties of the ingredients. Aquafaba contains soluble proteins and carbohydrates that provide water-holding capacity in bakery systems, as previously reported for its foaming, emulsifying, and water-absorption abilities [88,89]. In contrast, although soapwort extract is known for its foaming capacity due to its saponin content, it lacks documented evidence regarding water-binding functionality in muffin matrices.

Ash and fat contents also varied during storage. By day 30, CHC displayed the highest ash content (1.21%), while CC exhibited the greatest fat reduction (5.50%). In certain cases, apparent increases in fat content may have resulted from concentration effects due to moisture loss, leading to a higher proportion of solids, particularly lipids. Conversely, decreases in fat content can be attributed to lipid oxidation or hydrolysis, processes that are more likely to occur in bakery products rich in unsaturated fatty acids during storage [24,44]. Changes in ash content may also be attributed to similar concentration effects caused by moisture loss, as well as physical processes such as the redistribution or localized concentration of minerals within the muffin matrix over time [66]. Texture analysis showed a general increase in hardness, particularly in SWC and CHC, by day 15. Between day 0 and day 15, SWC exhibited a 319.9% increase in chewiness, rising from 529.96 to 2225.28, and a slight decrease in resilience (−0.91%). These marked changes in texture may be attributed to the foaming and structure-stabilizing potential of saponins present in soapwort extract, as previously reported in other food systems [15].

However, cohesiveness tended to decline across all samples, indicating partial structural weakening, possibly from starch retrogradation. Color parameters fluctuated throughout storage. Crust brightness (L*) remained highest in SWC, whereas crusts of BC and CHC darkened progressively. Internal crumb color also changed, with PC and FC showing decreased lightness and increased redness (a*), likely due to pigment-rich ingredients such as psyllium and flaxseed [19,67].

Aerobic plate counts (APC) ranged from 2.2 × 10^2^ to 2.1 × 10^3^ CFU/g and remained within the acceptable microbiological limits for ready-to-eat bakery products defined by the European Commission Regulation (EC) No. 2073/2005. *E. coli*, coliforms, *Staphylococcus aureus*, and yeast-mold counts stayed below the detection threshold (<10 CFU/g), and *Salmonella* spp. was not detected in any sample. These findings confirm that hygienic production was maintained and microbial safety ensured. Moreover, the inclusion of plant-based ingredients such as aquafaba, flaxseed, psyllium, and chia did not compromise microbial quality. In conclusion, the formulations demonstrated good shelf stability and remained safe for ambient storage over 30 days.

### 3.8. Sensorial Characteristics

**Figure 5:**
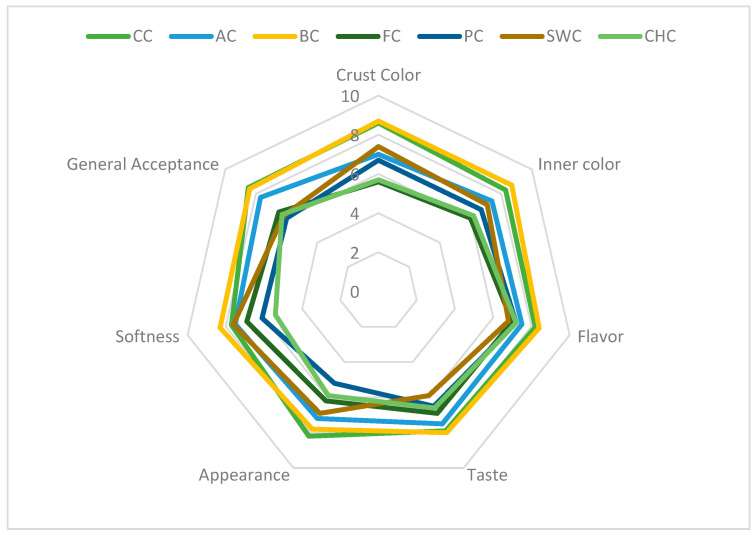
Radar diagram of sensory characteristics of muffin samples. CC: Control muffin, AC: Aquafaba muffin, BC: Banana muffin, FC: Flaxseed muffin, PC: Psyllium muffin, SWC: Soapwort muffin, CHC: Chia muffin.

Sensory characteristics are crucial in determining consumer acceptance of food products. The results of the sensory evaluation (n = 24) are illustrated in the radar diagram (Figure 5). The color characteristics of baked goods are influenced by both the inherent colors of the ingredients and the color changes resulting from their interactions during baking [90].

Crust and crumb color scores ranged from 5.60 to 8.70, with the BC sample receiving the highest ratings. The addition of mashed banana increased the sugar content in BC, likely enhancing visual appeal through intensified browning reactions [25]. A study of green banana flour-based cakes reported that higher banana content led to an increase in a* values [91], supporting this observation. Flavor scores ranged from 6.80 to 8.40, again with BC achieving the highest score. Although FC, PC, SWC, and CHC samples exhibited lower average scores for flavor and taste, the differences were not statistically significant (*p* > 0.05). Taste ratings for all muffins ranged from 5.90 to 8.00. Regarding appearance, the CC sample was rated highest (8.20), while the PC sample received the lowest score (5.20), likely due to its reduced b* value (Table 3). Softness ratings varied between 5.40 (CHC) and 8.30 (BC), with the lower score in CHC possibly resulting from the granular texture imparted by chia gel.

General acceptability scores ranged from 6.00 to 8.50. The BC sample closely approached the CC in overall preference, while FC, PC, SWC, and CHC showed no statistically significant differences (*p* > 0.05). Notably, all samples received scores above 6, indicating general acceptance by the panelists. It is widely acknowledged that achieving sensory acceptability comparable to traditional formulations can be challenging when using plant-based substitutes [63].

### 3.9. Chemometric Analysis

#### 3.9.1. Correlation Coefficient

**Figure 6:**
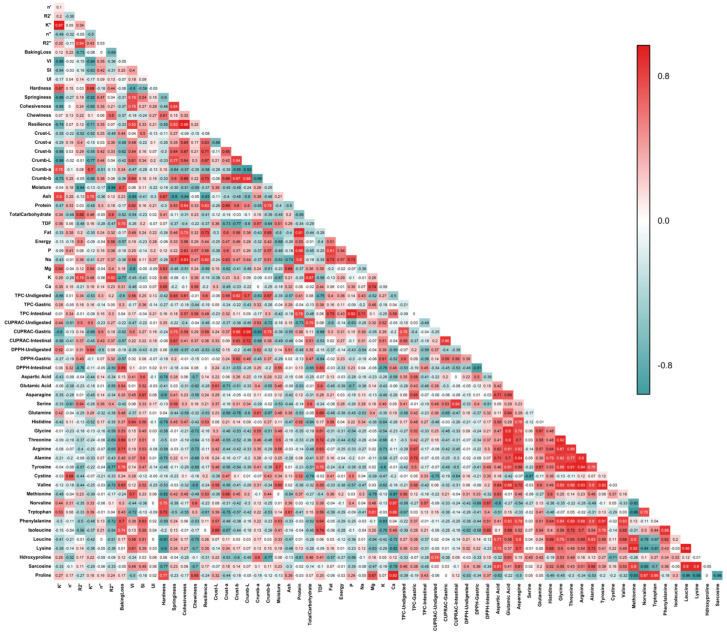
Pearson’s correlation heatmap of physicochemical, compositional, textural, and functional attributes of the samples.

The chemometric evaluation conducted in this study allowed for an integrated assessment of the physicochemical, textural, and functional properties of egg-free muffin formulations. Pearson correlation coefficients were calculated to examine the linear relationships among key variables, including physicochemical attributes, compositional parameters, textural metrics, and functional characteristics (Figure 6).

The strong positive correlations between protein content and texture parameters such as cohesiveness (R^2^ = 0.84) and resilience (R^2^ = 0.83) suggest that proteins significantly contribute to the structural integrity and elastic recovery of the muffin matrix. In particular, plant-derived hydrocolloids such as flaxseed and chia exhibit viscoelastic behavior similar to protein gels, thereby enhancing textural properties by improving moisture retention and providing structural support. Moreover, sodium ions may increase the ionic strength of the system, facilitating the formation of more organized gel networks in hydrocolloids such as chia and psyllium. In mucilage-rich systems such as chia, this effect can contribute to greater gel stability [19,20].

Protein content showed strong positive correlations with fat (R^2^ = 0.91), phosphorus (R^2^ = 0.89), and sodium (R^2^ = 0.80), which can be explained by the nutritional composition of the plant-based ingredients used in the muffin formulations. Ingredients such as chia, flaxseed, and chickpea are naturally rich in both protein and fat, while others, such as soapwort and psyllium, may contribute more to the mineral content. These correlations likely reflect the fact that certain nutrients are commonly found together within the same ingredient, rather than being influenced by separate formulation factors [92,93].

Glutamic acid exhibited strong positive correlations with threonine, glycine, and valine (0.88 ≤ R^2^ ≤ 0.90). Similarly, high correlations were observed among glycine, threonine, arginine, alanine, tyrosine, valine, and phenylalanine (0.80 ≤ R^2^ ≤ 0.93). This pattern reflects their natural presence in plant-based protein sources, such as flaxseed and aquafaba. Furthermore, amino acids such as phenylalanine, tyrosine, and glutamic acid exhibited positive correlations with protein and ash, supporting previous observations of co-occurrence in nutrient-dense muffin matrices [87].

Calcium showed strong positive correlations with proline, norvaline, and tryptophan (R^2^ = 0.93, 0.87, and 0.86, respectively). This pattern suggests that ingredients such as chickpea, chia, and soapwort extract naturally provide both calcium and amino acids, which are likely released together during digestion.

A strong positive correlation (R^2^ = 0.92) was observed between intestinal TPC and phosphorus content, as both phenolics and phosphorus are abundant in ingredients such as chia, flaxseed, and chickpea, and are released together during digestion. Total carbohydrate content was strongly correlated with potassium levels (R^2^ = 0.87). Ingredients such as banana and chickpea naturally contain both nutrients in high amounts, which explains the observed relationship. Total phenolic content (TPC), CUPRAC, and DPPH values were strongly correlated (0.88 ≤ R^2^ ≤ 0.86), reflecting synergistic antioxidant behavior, consistent with previous findings on legume- or fiber-enriched formulations [18,43].

The negative correlations (−0.75 ≤ R^2^ ≤ −0.91) observed between certain amino acids (e.g., asparagine, histidine, lysine, methionine) and hardness may be attributed to their potential interference with protein–polysaccharide interactions. As discussed by Yiasmin et al. [94], such amino acids can affect the electrostatic and hydrophilic balance within the gel matrix, potentially weakening network formation and resulting in softer textures.

Baking loss showed a negative correlation with moisture and total dietary fiber (TDF), underscoring the role of water-binding components in reducing mass loss during baking [54,61].

#### 3.9.2. Principal Component Analysis

**Figure 7:**
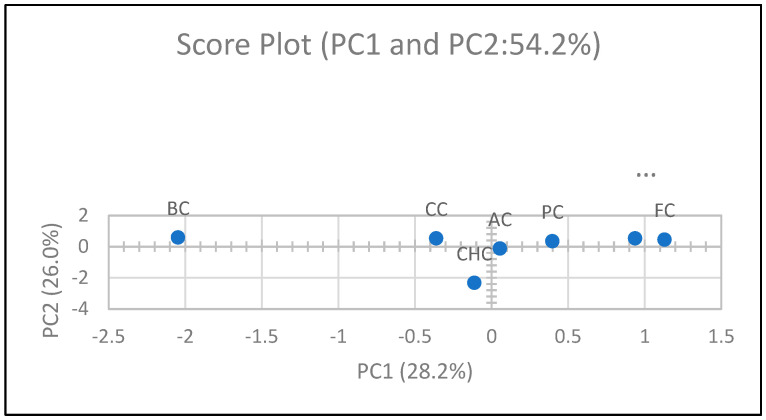
Principal component analysis (PCA) score plot of muffin samples. CC: Control muffin, AC: Aquafaba muffin, BC: Banana muffin, FC: Flaxseed muffin, PC: Psyllium muffin, SWC: Soapwort muffin, CHC: Chia muffin.

Principal component analysis (PCA) was conducted to uncover underlying data patterns, and the factor loadings are presented in Supplementary Table S3. The first two principal components (PC1: 28.2% and PC2: 26.0%) accounted for 54.2% of the total variance (Figure 7), indicating a sufficient level of dimensionality reduction to distinguish between sample groups. This analysis enabled the visualization of clustering behavior and differentiation between formulations based on their physicochemical and functional profiles.

PC1 was primarily associated with compositional and textural variables, including negative loadings for hardness (−0.489) and chewiness (−0.504), and positive loadings for baking loss (0.754), TDF (0.723), and amino acids such as arginine (0.914) and phenylalanine (0.976). These findings suggest that higher levels of amino acids and dietary fiber contributed to a softer texture and better moisture retention. This trend aligns with the known functionality of hydrocolloid-rich ingredients such as chia and flaxseed, which enhance water-holding capacity and promote a more tender crumb structure [19,20].

PC2 accounted for 26.0% of the total variance and primarily explained variation in antioxidant and mineral-related features. High positive loadings for lysine (0.930), leucine (0.830), and methionine (0.949) contrasted with negative contributions from calcium (−0.956), magnesium (−0.706), and hardness (−0.757). This axis appears to distinguish formulations characterized by elevated levels of essential amino acids—associated with improved nutritional value and softer textures—from those with higher mineral concentrations, which were linked to increased firmness. This interpretation aligns with earlier correlation patterns, suggesting that mineral-rich formulations tended to display more rigid structural attributes.

**Figure 8:**
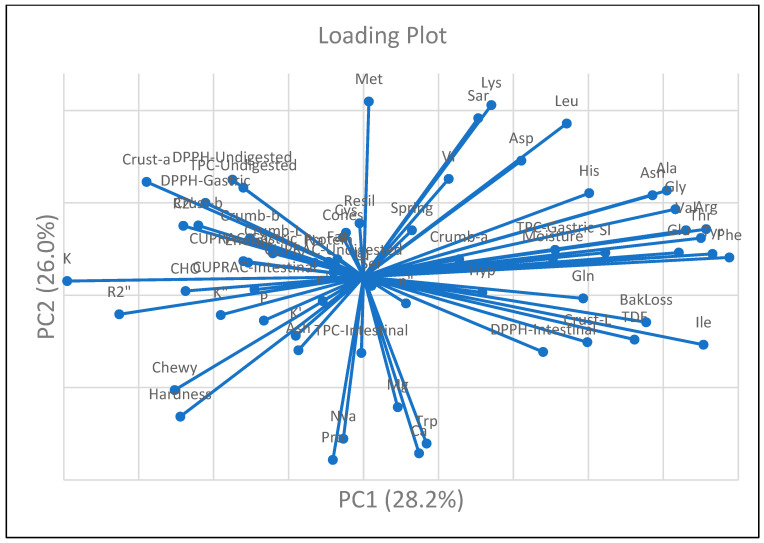
Principal component analysis (PCA) loading plot of muffin samples.

The loading plot (Figure 8) confirmed that PC1 was driven by compositional and textural variables, while PC2 was shaped by differences in bioactive components such as TPC, CUPRAC, and DPPH, and essential amino acids, supporting earlier findings [95,96].

PC3 (15.0%) primarily accounted for differences in color and antioxidant parameters. High positive loadings were observed for crumb lightness (L*) (0.925), gastric CUPRAC activity (0.928), and crust yellowness (b*) (0.741), while crumb redness (a*) loaded negatively (−0.916). These results suggest that PC3 reflects variations in crumb brightness and antioxidant activity under gastric conditions.

PC4 (11.0%) and PC5 (7.2%) appeared to describe finer distinctions related to structural resilience, the behavior of phenolics during digestion, and carbohydrate levels. PC4, in particular, showed strong positive contributions from protein (0.813) and intestinal TPC (0.856), implying that protein and phenolic compounds may be released simultaneously during digestion. This supports the idea that certain nutrients, such as proteins and phenolics, are naturally occurring in plant-based matrices and tend to become bioaccessible together [95,96].

The score plot (Figure 7) supported the PCA findings, with FC, PC, and AC samples clustering positively on PC1, reflecting their high fiber and amino acid content. In contrast, BC and CC were positioned along PC2, highlighting their elevated antioxidant potential. CHC appeared as an outlier across both axes, indicating distinctive structural and functional characteristics that set it apart from the other formulations. This distinct positioning may stem from the limited protein contribution and differing gelation behavior of chia gel compared to whole egg, potentially leading to altered structural integrity and functional outcomes [20].

#### 3.9.3. Hierarchical Cluster Analysis

**Figure 9:**
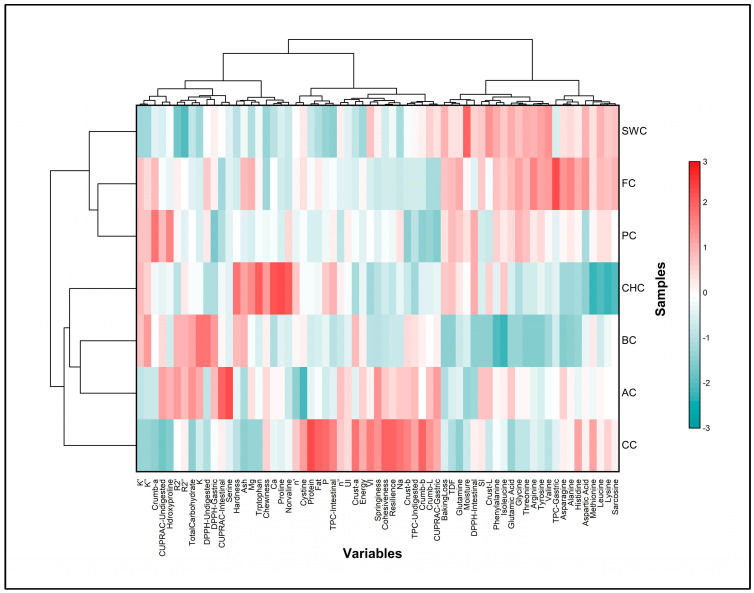
Cluster map of muffin samples.

Hierarchical cluster analysis (HCA) is a multivariate statistical technique used to identify similarities and differences by dividing samples into clusters based on their shared characteristics. By applying Ward’s method with Euclidean distance, a dendrogram was constructed to visualize the structural and functional relationships among the samples (Figure 9). This method produces a dendrogram that allows visual interpretation of how closely related samples are across multiple variables. This approach effectively complemented the PCA findings, revealing both broad groupings and formulation-specific divergences [97].

Two primary clusters emerged: Cluster I included FC, PC, and AC samples, all of which shared compositional features such as higher amino acid content, moisture, and dietary fiber. These components were also linked to favorable textural parameters (e.g., lower hardness), suggesting improved structural integrity. As shown in earlier studies, the presence of hydrocolloid-rich components in aquafaba- and fiber-based formulations tends to improve moisture retention and soften the muffin matrix—explaining the grouping pattern observed in Cluster I [7,11,98].

Cluster II, occupied solely by the SWC sample, formed a distinct subgroup with intermediate characteristics. Its unique position may reflect the role of soapwort extract, which is known for its mineral and saponin content, contributing simultaneously to antioxidant potential and textural modification [15]. BC and CHC branched separately from the main clusters, indicating unique formulation behavior. Its relatively low protein content and dense mucilaginous structure may have limited its interaction with other matrix components, resulting in weaker structural integration compared to other egg alternatives such as aquafaba or flaxseed gel [20]. BC, on the other hand, showed elevated antioxidant activity (DPPH, CUPRAC) and mineral content, likely linked to banana’s natural composition. Its functional outlier status reinforces the idea that certain ingredients can drive specific sample clustering due to their dominant compositional effects [18,91].

Together, the PCA and HCA results revealed the multidimensional differentiation among the formulations. These findings support the strategic use of aquafaba and fiber-rich ingredients in improving both structural and functional aspects of vegan muffin formulations. Such multivariate approaches serve as effective tools for guiding product optimization in egg-free bakery systems, aligning with previous work emphasizing legume-derived protein and polysaccharide synergies [4,6,16,54].

## 4. Conclusions

This study provides a comprehensive and comparative evaluation of widely used plant-based egg substitutes in vegan muffin formulations. Among the tested alternatives, ripe banana and aquafaba emerged as the most promising in balancing structure and consumer acceptability, while chia and psyllium significantly enhanced nutritional and antioxidant properties. Notably, banana-based samples achieved the highest sensory scores, offering a viable clean-label solution for consumer-preferred textures and flavors. The study also revealed that plant-based substitutes affect rheological behavior differently; high G′ values in banana and chia samples were associated with denser batters and reduced volume indices, while soapwort extract improved foaming without compromising crumb softness. Nutritionally, the use of flaxseed and psyllium resulted in marked increases in dietary fiber and mineral content, aligning with current trends in functional bakery products.

This work fills a critical gap by systematically analyzing not only the physical and sensory effects of egg substitutes but also their impact on amino acid composition and bioaccessible phenolic content. Importantly, the extended shelf-life stability of selected formulations (demonstrated by maintained microbiological safety and physicochemical integrity over 30 days) adds practical value for industrial applications. From an industrial perspective, the findings offer a science-backed formulation framework for developing vegan bakery products that meet nutritional regulations, sustainability goals, and consumer expectations.

Recognizing the unique functional roles of individual ingredients is essential for rational formulation design in plant-based baking, and the present study offers a practical, evidence-based framework for both researchers and product developers. Furthermore, this study highlights that plant-based egg substitutes substantially alter the textural characteristics of muffins, emphasizing the importance of optimizing formulations according to the desired texture profile. This perspective underscores the need for targeted formulation strategies that balance technological functionality, nutrition, and consumer expectations in plant-based bakery products.

Future research should expand on these findings by conducting large-scale consumer acceptance tests across different demographic groups and markets to validate sensory preferences. Moreover, detailed profiling of aroma compounds and potential process-derived contaminants is warranted to ensure comprehensive product safety and quality. Investigations into the in vivo bioavailability of phenolic compounds and long-term nutritional effects would further support health claims. In addition, the potential benefits of combining two or more egg substitutes in a single formulation merit further investigation, as such combinations may offer complementary advantages in terms of protein content, flavor, texture, and overall product performance. Implementing this approach in future studies could help develop optimized vegan muffin formulations with improved technological, nutritional, and sensory qualities.

## Figures and Tables

**Table 1:** Formulation of muffin samples.

Ingredients ^1^ (g)	CC	AC	BC	FC	PC	SWC	CHC
Wheat flour	200 g	200 g	200 g	200 g	200 g	200 g	200 g
Sunflower oil	100 g	100 g	100 g	100 g	100 g	100 g	100 g
Whole egg	90 g	0	0	0	0	0	0
Aquafaba (chickpea)	0	90 g	0	0	0	0	0
Banana	0	0	90 g	0	0	0	0
Flaxseed gel (FG)	0	0	0	90 g	0	0	0
Psyllium gel (PG)	0	0	0	0	90 g	0	0
Soapwort extract mixture (SM)	0	0	0	0	0	90 g	0
Chia gel (CG)	0	0	0	0	0	0	90 g
Sugar	150 g	150 g	150 g	150 g	150 g	150 g	150 g
Almond milk	0	32 g	32 g	32 g	32 g	32 g	32 g
Cow milk	32 g	0	0	0	0	0	0
Baking powder	5 g	5 g	5 g	5 g	5 g	5 g	5 g
Vanillin	3 g	3 g	3 g	3 g	3 g	3 g	3 g

^1^ Ingredients at 21 ± 1 °C; CC: Control muffin, AC: Aquafaba muffin, BC: Banana muffin, FC: Flaxseed muffin, PC: Psyllium muffin, SWC: Soapwort muffin, CHC: Chia muffin; CG: Chia gel, PG: Psyllium seed powder gel, FG: Flaxseed powder gel, SM: Soapwort extract mixture.

**Table 2:** Dynamic shear parameters of power-law functions describing the G′ and G′′ values of muffin samples.

	$G^{\prime }=K^{\prime }{\left(\omega \right)}^{n^{\prime }}$			$G^{\prime \prime }=K^{\prime \prime }{\left(\omega \right)}^{n^{\prime \prime }}$			tan δ
Samples	K’ (Pa.s*^n^*)	n’	R^2^	K” (Pa.s*^n^*)	n”	R^2^	
CC	598.32 ± 17.49 ^c^	0.458 ± 0.07 ^a^	0.942	532.25 ± 23.31 ^d^	0.533 ± 0.12 ^a^	0.937	0.845 ± 0.02 ^a^
AC	1235.10 ± 29.01 ^b^	0.295 ± 0.05 ^d^	0.989	970.55 ± 15.82 ^c^	0.420 ± 0.14 ^b^	0.959	0.706 ± 0.01 ^c^
BC	3090.70 ± 38.97 ^a^	0.365 ± 0.04 ^bc^	0.978	2268.70 ± 21.68 ^a^	0.405 ± 0.18 ^b^	0.961	0.707 ± 0.00 ^c^
FC	3065.00 ± 24.64 ^a^	0.307 ± 0.08 ^c^	0.950	1751.30 ± 19.70 ^b^	0.386 ± 0.16 ^c^	0.931	0.551 ± 0.01 ^e^
PC	3107.00 ± 13.12 ^a^	0.336 ± 0.02 ^c^	0.946	1906.60 ± 27.22 ^a^	0.375 ± 0.13 ^c^	0.919	0.598 ± 0.00 ^d^
SWC	818.10 ± 13.12 ^b^	0.383 ± 0.02 ^b^	0.879	604.89 ± 12.55 ^d^	0.436 ± 0.09 ^b^	0.872	0.748 ± 0.01 ^b^
CHC	3117.50 ± 41.16 ^a^	0.396 ± 0.01 ^b^	0.908	1782.50 ± 20.47 ^b^	0.379 ± 0.11 ^c^	0.940	0.621 ± 0.01 ^d^

Results are displayed as the mean ± standard deviation. Means followed by different letters within a column are significantly different (*p* < 0.05). tan δ: Loss factor at 1.36 rad s^−1^. CC: Control muffin, AC: Aquafaba muffin, BC: Banana muffin, FC: Flaxseed muffin, PC: Psyllium muffin, SWC: Soapwort muffin, CHC: Chia muffin.

**Table 3:** Physical properties of muffin samples.

		CC	AC	BC	FC	PC	SWC	CHC
Volumetric properties								
Baking Loss (%)		6.41 ± 0.39 ^bc^	5.63 ± 1.03 ^c^	4.83 ± 0.63 ^c^	8.91 ± 0.16 ^a^	8.16 ± 0.64 ^ab^	9.23 ± 1.00 ^a^	8.67 ± 1.61 ^ab^
VI (mm)		119.00 ± 3.61 ^a^	100.67 ± 5.13 ^bc^	88.33 ± 4.93 ^d^	93.00 ± 1.00 ^cd^	98.33 ± 2.08 ^c^	108.00 ± 1.00 ^b^	84.00 ± 2.65 ^d^
SI (mm)		9.00 ± 1.00 ^ab^	10.33 ± 1.53 ^a^	4.67 ± 0.58 ^b^	10.00 ± 1.00 ^a^	6.67 ± 2.08 ^ab^	10.00 ± 2.65 ^a^	7.00 ± 1.00 ^ab^
UI (mm)		1.00 ± 1.00 ^a^	1.00 ± 1.73 ^a^	0.00 ± 1.00 ^a^	0.00 ± 1.73 ^a^	0.67 ± 1.53 ^a^	0.00 ± 1.00 ^a^	0.33 ± 0.58 ^a^
Texture								
Hardness (gf)		1284.74 ± 104.18 ^d^	1584.69 ± 141.60 ^d^	2220.74 ± 110.34 ^b^	1435.25 ± 77.01 ^d^	1747.63 ± 199.09 ^c^	1266.15 ± 62.22 ^d^	2735.73 ± 76.35 ^a^
Springiness		0.91 ± 0.01 ^a^	0.88 ± 0.02 ^a^	0.72 ± 0.03 ^c^	0.74 ± 0.03 ^c^	0.76 ± 0.02 ^c^	0.81 ± 0.02 ^b^	0.73 ± 0.01 ^c^
Cohesiveness		0.71 ± 0.02 ^a^	0.60 ± 0.03 ^b^	0.50 ± 0.01 ^c^	0.51 ± 0.06 ^c^	0.55 ± 0.01 ^bc^	0.52 ± 0.01 ^c^	0.51 ± 0.02 ^c^
Chewiness		828.54 ± 70.46 ^bc^	832.87 ± 116.75 ^bc^	799.28 ± 75.46 ^bc^	543.42 ± 35.63 ^d^	733.84 ± 73.53 ^c^	529.96 ± 35.35 ^d^	1012.27 ± 21.87 ^a^
Resilience		0.33 ± 0.04 ^a^	0.26 ± 0.020 ^ab^	0.19 ± 0.05 ^b^	0.21 ± 0.06 ^b^	0.22 ± 0.01 ^b^	0.22 ± 0.01 ^b^	0.20 ± 0.01 ^b^
Color								
Crust	L*	58.98 ± 3.05 ^c^	69.12 ± 1.96 ^ab^	56.17 ± 3.52 ^c^	63.45 ± 4.34 ^b^	58.18 ± 2.81 ^c^	73.17 ± 1.31 ^a^	68.15 ± 2.00 ^ab^
	a*	12.47 ± 1.88 ^a^	3.21 ± 1.83 ^c^	8.80 ± 2.31 ^b^	2.82 ± 0.78 ^d^	4.48 ± 0.36 ^c^	1.64 ± 0.85 ^e^	0.38 ± 0.87 ^f^
	b*	30.99 ± 2.86 ^a^	25.92 ± 2.26 ^b^	24.16 ± 1.03 ^b^	16.58 ± 1.80 ^d^	12.15 ± 1.16 ^e^	21.29 ± 1.24 ^c^	17.01 ± 2.03 ^d^
	ΔE	-	18.14 ± 0.75 ^a^	9.44 ± 0.80 ^b^	23.42 ± 0.35 ^a^	22.71 ± 0.92 ^a^	19.97 ± 1.02 ^a^	23.55 ± 0.20 ^a^
	C*	33.87 ± 2.95 ^a^	26.20 ± 2.38 ^b^	26.47 ± 0.82 ^b^	17.30 ± 0.47 ^c^	13.21 ± 0.88 ^d^	17.90 ± 0.70 ^c^	21.17 ± 0.12 ^c^
	h°	68.41 ± 4.16 ^c^	83.05 ± 0.42 ^ab^	70.86 ± 3.32 ^c^	88.76 ± 0.04 ^a^	70.54 ± 1.37 ^c^	81.17 ± 0.83 ^b^	85.57 ± 0.04 ^ab^
Crumb	L*	75.03 ± 0.84 ^a^	71.43 ± 1.24 ^b^	66.43 ± 0.91 ^c^	61.26 ± 1.27 ^d^	59.45 ± 0.66 ^d^	69.67 ± 1.76 ^b^	64.39 ± 1.59 ^c^
	a*	−2.27 ± 0.29 ^f^	−0.99 ± 0.16 ^e^	0.44 ± 0.43 ^c^	1.97 ± 0.29 ^b^	3.68 ± 0.14 ^a^	−0.45 ± 0.30 ^d^	−0.03 ± 0.42 ^cd^
	b*	24.32 ± 0.80 ^a^	16.48 ± 1.10 ^b^	16.85 ± 0.39 ^b^	13.82 ± 0.37 ^c^	10.56 ± 0.18 ^d^	17.26 ± 0.18 ^b^	14.67 ± 1.36 ^c^
	ΔE	-	8.38 ± 1.58 ^de^	10.65 ± 0.91 ^d^	13.36 ± 0.93 ^c^	20.70 ± 0.73 ^a^	17.49 ± 0.25 ^b^	7.88 ± 0.41 ^e^
	C*	24.39 ± 0.86 ^a^	16.08 ± 0.73 ^b^	16.87 ± 0.37 ^b^	14.74 ± 0.33 ^c^	11.16 ± 0.03 ^d^	13.92 ± 0.08 ^c^	17.24 ± 0.04 ^b^
	h°	275.15 ± 0.10 ^a^	273.27 ± 0.51 ^a^	88.55 ± 0.12 ^b^	270.13 ± 0.02 ^a^	71.05 ± 0.57 ^b^	81.94 ± 0.20 ^b^	271.51 ± 0.15 ^a^

Results are displayed as the mean ± standard deviation. Means followed by different letters within a line are significantly different (*p* < 0.05). CC: Control muffin, AC: Aquafaba muffin, BC: Banana muffin, FC: Flaxseed muffin, PC: Psyllium muffin, SWC: Soapwort muffin, CHC: Chia muffin; VI: Volume index, SI: Symmetry index, UI: Uniformity index.

**Table 4:** Chemical composition (g/100 g) and mineral composition (mg/1000 g) of muffin samples.

	**Moisture**	**Ash**	**Protein**	**Fat**	**TDF**	**Total Carbohydrate**	**Energy (kcal/100 g)**
CC	14.46 ± 0.09 ^d^	0.71 ± 0.04 ^g^	8.54 ± 0.04 ^a^	22.16 ± 0.351 ^a^	0.73 ± 0.15 ^c^	53.53 ± 0.35 ^d^	448.53 ± 10.86 ^a^
AC	13.58 ± 0.08 ^e^	0.75 ± 0.06 ^f^	5.65 ± 0.08 ^c^	19.80 ± 0.457 ^bc^	0.93 ± 0.21 ^bc^	59.00 ± 0.30 ^a^	440.27 ± 12.11 ^ab^
BC	14.90 ± 0.09 ^d^	1.03 ± 0.05 ^b^	5.37 ± 0.09 ^e^	19.37 ± 0.612 ^bc^	0.60 ± 0.30 ^c^	58.60 ± 0.30 ^a^	434.77 ± 12.43 ^ab^
FC	15.89 ± 0.10 ^c^	1.02 ± 0.02 ^c^	5.89 ± 0.09 ^b^	20.32 ± 0.454 ^b^	1.50 ± 0.10 ^a^	55.40 ± 0.33 ^bc^	433.70 ± 10.31 ^ab^
PC	16.40 ± 0.34 ^b^	0.84 ± 0.02 ^e^	5.52 ± 0.02 ^d^	19.34 ± 0.405 ^bc^	1.50 ± 0.26 ^a^	56.23 ± 0.35 ^b^	426.33 ± 9.58 ^ab^
SWC	19.06 ± 0.17 ^a^	0.86 ± 0.05 ^d^	5.24 ± 0.06 ^f^	18.78 ± 0.353 ^c^	1.30 ± 0.20 ^ab^	54.70 ± 0.40 ^c^	414.87 ± 13.39 ^b^
CHC	15.76 ± 0.06 ^c^	1.06 ± 0.06 ^a^	5.92 ± 0.03 ^b^	19.72 ± 0.401 ^bc^	1.47 ± 0.25 ^ab^	55.97 ± 0.35 ^b^	428.93 ± 9.87 ^ab^
	P		Na		Mg	K	Ca
CC	2188.36 ± 103.85 ^a^		2001.69 ± 121.29 ^a^		98.99 ± 9.98 ^g^	747.14 ± 25.31 ^e^	219.02 ± 1.16 ^c^
AC	1700.91 ± 31.98 ^c^		1838.19 ± 33.94 ^b^		158.80 ± 5.03 ^c^	1169.46 ± 15.75 ^b^	244.99 ± 0.66 ^b^
BC	1757.49 ± 5.56 ^c^		1664.06 ± 10.56 ^d^		143.37 ± 2.45 ^d^	1352.42 ± 3.13 ^a^	178.61 ± 1.59 ^d^
FC	1771.93 ± 1.83 ^c^		1668.81 ± 5.59 ^d^		188.12 ± 0.40 ^b^	775.48 ± 6.15 ^d^	234.96 ± 0.56 ^b^
PC	1764.91 ± 9.46 ^c^		1804.63 ± 5.84 ^bc^		127.90 ± 1.15 ^e^	776.26 ± 4.77 ^d^	231.90 ± 9.01 ^b^
SWC	1569.93 ± 6.29 ^d^		1606.52 ± 2.77 ^d^		110.62 ± 1.53 ^f^	600.01 ± 3.16 ^f^	185.86 ± 0.33 ^d^
CHC	1944.11 ± 0.22 ^b^		1711.41 ± 1.44 ^cd^		208.73 ± 1.84 ^a^	872.31 ± 0.59 ^c^	372.00 ± 13.85 ^a^

Results are displayed as the mean ± standard deviation. Means followed by different letters within a column are significantly different (*p* < 0.05). CC: Control muffin, AC: Aquafaba muffin, BC: Banana muffin, FC: Flaxseed muffin, PC: Psyllium muffin, SWC: Soapwort muffin, CHC: Chia muffin.

**Table 5:** Total phenolic content, antioxidant activities, and bioaccessibility of muffin samples.

	Undigested	Simulated Gastric Digestion	Simulated Intestinal Digestion	Bioaccessibility (%)
TPC (mg GAE/g)				
CC	0.083 ± 0.004 ^a,B^	0.083 ± 0.001 ^b,B^	0.140 ± 0.001 ^a,A^	168.675
AC	0.094 ± 0.048 ^a,A^	0.093 ± 0.001 ^b,A^	0.087 ± 0.001 ^e,A^	92.553
BC	0.066 ± 0.007 ^a,A^	0.076 ± 0.002 ^b,A^	0.073 ± 0.001 ^f,A^	111.606
FC	0.049 ± 0.026 ^a,B^	0.154 ± 0.012 ^a,A^	0.097 ± 0.001 ^d,AB^	197.959
PC	0.029 ± 0.022 ^a,A^	0.085 ± 0.003 ^b,B^	0.105 ± 0.001 ^c,B^	362.069
SWC	0.059 ± 0.063 ^a,A^	0.079 ± 0.002 ^b,A^	0.062 ± 0.001 ^g,A^	105.084
CHC	0.031 ± 0.017 ^a,B^	0.084 ± 0.015 ^b,AB^	0.125 ± 0.002 ^b,A^	403.226
CUPRAC (µmol TE/g)				
CC	0.020 ± 0.008 ^a,C^	0.884 ± 0.007 ^a,A^	0.633 ± 0.015 ^b,B^	3165
AC	0.137 ± 0.011 ^a,C^	0.917 ± 0.011 ^a,B^	1.016 ± 0.009 ^a,A^	741.605
BC	0.110 ± 0.014 ^a,C^	0.710 ± 0.012 ^c,A^	0.627 ± 0.017 ^b,B^	570
FC	0.088 ± 0.009 ^a,C^	0.452 ± 0.012 ^e,A^	0.363 ± 0.023 ^d,B^	412.500
PC	0.148 ± 0.094 ^a,B^	0.387 ± 0.013 ^f,A^	0.387 ± 0.012 ^d,A^	261.486
SWC	0.071 ± 0.010 ^a,C^	0.757 ± 0.016 ^b,A^	0.601 ± 0.013 ^bc,B^	846.478
CHC	0.076 ± 0.007 ^a,C^	0.604 ± 0.003 ^d,A^	0.557 ± 0.017 ^c,B^	732.894
DPPH (µmol TE/g)				
CC	48.223 ± 3.217 ^d,A^	35.048 ± 1.191 ^cd,B^	1.584 ± 0.005 ^c,C^	3.284
AC	50.893 ± 1.641 ^d,A^	52.509 ± 1.203 ^ab,A^	1.190 ± 0.002 ^e,B^	2.338
BC	85.599 ± 1.905 ^a,A^	58.560 ± 2.195 ^a,B^	1.134 ± 0.001 ^f,C^	1.324
FC	72.292 ± 0.185 ^b,A^	39.065 ± 10.666 ^bcd,B^	1.416 ± 0.005 ^d,C^	1.958
PC	65.263 ± 3.203 ^bc,A^	12.913 ± 0.521 ^e,B^	1.823 ± 0.012 ^a,C^	2.793
SWC	61.362 ± 0.297 ^c,A^	41.398 ± 1.960 ^abc,B^	1.672 ± 0.012 ^b,C^	2.724
CHC	47.510 ± 1.549 ^d,A^	23.306 ± 2.915 ^de,B^	1.828 ± 0.005 ^a,C^	3.847

Different lowercase letters in the columns based on the Tukey test indicate a significant difference (*p* < 0.05) between TPC, CUPRAC, and DPPH of muffin samples, while different uppercase letters for the line based on the Tukey test indicate a significant difference (*p* < 0.05) between the total phenolic content of muffin samples at different digestion phases. CC: Control muffin, AC: Aquafaba muffin, BC: Banana muffin, FC: Flaxseed muffin, PC: Psyllium muffin, SWC: Soapwort muffin, CHC: Chia muffin.

**Table 6:** Total amino acid profile of muffin samples (g/100 g).

Amino Acids	CC	AC	BC	CHC	PC	FC	SWC
Acidic amino acids							
Aspartic Acid	0.065 ± 0.003 ^c^	0.067 ± 0.003 ^c^	0.056 ± 0.002 ^d^	0.030 ± 0.001 ^e^	0.038 ± 0.001 ^e^	0.094 ± 0.004 ^a^	0.083 ± 0.003 ^b^
Glutamic Acid	0.476 ± 0.020 ^d^	0.873 ± 0.025 ^b^	0.474 ± 0.022 ^d^	0.623 ± 0.018 ^c^	0.845 ± 0.020 ^b^	0.926 ± 0.022 ^a^	0.924 ± 0.021 ^a^
Basic amino acids							
Arginine	1.007 ± 0.030 ^e^	1.035 ± 0.035 ^d^	0.832 ± 0.030 ^g^	0.970 ± 0.035 ^f^	1.193 ± 0.045 ^c^	1.455 ± 0.050 ^a^	1.314 ± 0.040 ^b^
Histidine	0.321 ± 0.015 ^b^	0.277 ± 0.020 ^d^	0.236 ± 0.018 ^f^	0.248 ± 0.020 ^e^	0.276 ± 0.015 ^d^	0.328 ± 0.018 ^a^	0.295 ± 0.016 ^c^
Lysine	0.114 ± 0.004 ^b^	0.094 ± 0.003 ^d^	0.082 ± 0.003 ^e^	0.008 ± 0.001 ^f^	0.105 ± 0.004 ^c^	0.125 ± 0.005 ^a^	0.118 ± 0.004 ^b^
Neutral amino acids							
Alanine	0.136 ± 0.005 ^c^	0.131 ± 0.004 ^c^	0.104 ± 0.004 ^e^	0.110 ± 0.005 ^d^	0.135 ± 0.006 ^c^	0.159 ± 0.007 ^a^	0.144 ± 0.006 ^b^
Serine	<LOD	0.004 ± 0.001 ^a^	<LOD	<LOD	<LOD	<LOD	<LOD
Asparagine	0.072 ± 0.004 ^b^	0.071 ± 0.003 ^b^	0.049 ± 0.003 ^e^	0.054 ± 0.002 ^d^	0.067 ± 0.003 ^c^	0.079 ± 0.004 ^a^	0.070 ± 0.003 ^b^
Glutamine	0.007 ± 0.001 ^e^	0.011 ± 0.002 ^b^	0.008 ± 0.001 ^d^	0.009 ± 0.002 ^c^	0.012 ± 0.002 ^a^	0.013 ± 0.002 ^a^	0.011 ± 0.002 ^b^
Glycine	0.039 ± 0.002 ^d^	0.054 ± 0.003 ^c^	0.030 ± 0.002 ^e^	0.037 ± 0.003 ^d^	0.062 ± 0.004 ^b^	0.071 ± 0.005 ^a^	0.064 ± 0.004 ^b^
Threonine	0.050 ± 0.003 ^c^	0.069 ± 0.004 ^b^	0.039 ± 0.002 ^d^	0.057 ± 0.003 ^c^	0.084 ± 0.005 ^a^	0.087 ± 0.005 ^a^	0.088 ± 0.005 ^a^
Tyrosine	0.048 ± 0.003 ^c^	0.048 ± 0.003 ^c^	0.037 ± 0.003 ^d^	0.050 ± 0.003 ^c^	0.062 ± 0.004 ^b^	0.068 ± 0.004 ^a^	0.069 ± 0.004 ^a^
Cystine	0.061 ± 0.004 ^a^	0.017 ± 0.002 ^d^	0.045 ± 0.003 ^b^	0.042 ± 0.003 ^c^	0.041 ± 0.002 ^c^	0.046 ± 0.003 ^b^	0.046 ± 0.003 ^b^
Valine	0.124 ± 0.006 ^d^	0.138 ± 0.007 ^c^	0.109 ± 0.005 ^e^	0.124 ± 0.006 ^d^	0.151 ± 0.008 ^b^	0.177 ± 0.009 ^a^	0.171 ± 0.008 ^a^
Methionine	0.088 ± 0.004 ^a^	0.053 ± 0.003 ^d^	0.062 ± 0.004 ^c^	<LOD	0.061 ± 0.003 ^c^	0.067 ± 0.003 ^b^	0.065 ± 0.003 ^b^
Norvaline	0.004 ± 0.001 ^c^	<LOD	0.001 ± 0.000 ^d^	0.018 ± 0.001 ^a^	0.007 ± 0.001 ^b^	<LOD	<LOD
Tryptophan	0.007 ± 0.001 ^e^	0.019 ± 0.002 ^b^	0.011 ± 0.002 ^d^	0.037 ± 0.003 ^a^	0.018 ± 0.002 ^b^	0.019 ± 0.002 ^b^	0.016 ± 0.002 ^c^
Phenylalanine	0.105 ± 0.004 ^d^	0.124 ± 0.005 ^c^	0.014 ± 0.002 ^e^	0.098 ± 0.005 ^d^	0.144 ± 0.007 ^b^	0.155 ± 0.007 ^a^	0.159 ± 0.007 ^a^
Isoleucine	0.087 ± 0.003 ^e^	0.107 ± 0.004 ^d^	<LOD	0.134 ± 0.006 ^a^	0.119 ± 0.005 ^c^	0.127 ± 0.005 ^b^	0.131 ± 0.005 ^a^
Leucine	0.330 ± 0.010 ^e^	0.339 ± 0.011 ^d^	0.270 ± 0.008 ^f^	0.143 ± 0.005 ^g^	0.357 ± 0.012 ^c^	0.408 ± 0.013 ^a^	0.398 ± 0.012 ^b^
Hydroxyproline	<LOD	0.065 ± 0.005 ^b^	0.020 ± 0.002 ^e^	0.035 ± 0.003 ^c^	0.083 ± 0.005 ^a^	0.032 ± 0.003 ^d^	0.036 ± 0.003 ^c^
Sarcosine	0.052 ± 0.003 ^c^	0.052 ± 0.003 ^c^	0.051 ± 0.003 ^c^	0.037 ± 0.002 ^d^	0.054 ± 0.004 ^b^	0.057 ± 0.004 ^a^	0.055 ± 0.004 ^b^
Proline	0.037 ± 0.002 ^c^	0.071 ± 0.004 ^b^	0.034 ± 0.003 ^c^	0.217 ± 0.008 ^a^	0.028 ± 0.002 ^d^	0.025 ± 0.002 ^e^	0.027 ± 0.002 ^d^

Results are displayed as the mean ± standard deviation. Means followed by different letters within a line are significantly different (*p* < 0.05). “<LOD”: below the limit of detection. CC: Control muffin, AC: Aquafaba muffin, BC: Banana muffin, FC: Flaxseed muffin, PC: Psyllium muffin, SWC: Soapwort muffin, CHC: Chia muffin.

## Data Availability

The original contributions presented in this study are included in the article. Further inquiries can be directed to the corresponding author.

## Supplementary Materials

The following supporting information can be downloaded at: https://www.mdpi.com/article/10.3390/foods14173012/s1. Supplementary Table S1. Changes in physicochemical properties of muffin samples throughout shelf life. Supplementary Table S2. Microbiological analysis results of muffin samples throughout shelf life. Supplementary Table S3. Explained variance of the first seven principal components.
