# Supplementary material for: Evaluation of Plant-Based Egg Substitutes in Vegan Muffins: Functional, Structural, and Nutritional Characterization
Source: Foods. 2025 Aug 28;14(17):3012. doi: 10.3390/foods14173012 (PMC12428069; doi:10.3390/foods14173012)
Supplement: Supplementary file 1 [file foods-14-03012-s001.zip › foods-3815602-supplementary.pdf]

Supplementary Materials:

Supplementary Table S1. Changes in physicochemical properties of muffin samples throughout shelf life

		CC	AC	BC	FC	PC	SWC	CHC
	**Chemical properties**							
	Day 0	14.46±0.09^{e}	13.58±0.08^{f}	14.90±0.09^{d}	15.89±0.10^{c}	16.40±0.34^{b}	19.06±0.01^{a}	15.76±0.06^{c}
Moisture (%)	Day 15	12.10±0.29^{de}	13.26±0.45^{bc}	13.12±0.26^{cd}	15.17±0.33^{ab}	15.87±0.45^{a}	15.72±0.47^{a}	11.55±0.31^{e}
	Day 30	10.07±0.02^{e}	12.16±0.05^{a}	10.65±0.05^{c}	9.07±0.026^{g}	11.66±0.06^{b}	9.22±0.02^{f}	10.27±0.02^{d}
	Day 0	0.71±0.04^{d}	0.75±0.06^{d}	1.03±0.05^{b}	1.02±0.02^{b}	0.84±0.02^{c}	0.86±0.01^{c}	1.06±0.06^{a}
Ash (%)	Day 15	0.92±0.04^{a}	0.82±0.46^{ab}	0.71±0.60^{bc}	0.74±0.41^{bc}	0.74±0.51^{bc}	0.64±0.34^{c}	0.69±0.45^{c}
	Day 30	0.83±0.02^{b}	1.15±0.02^{a}	0.72±0.03^{c}	0.57±0.02^{d}	0.57±0.02^{d}	0.82±0.03^{b}	1.21±0.04^{a}
	Day 0	22.16±0.35^{a}	19.80±0.046^{c}	19.37±0.61^{d}	20.32±0.45^{b}	19.34±0.41^{d}	18.78±0.35^{e}	19.72±0.40^{c}
Fat (%)	Day 15	20.32±0.45^{a}	18.76±0.61^{bc}	18.10±0.46^{c}	20.01±0.50^{ab}	19.79±0.55^{ab}	19.40±0.46^{abc}	18.33±0.40^{c}
	Day 30	16.66±0.07^{f}	21.04±0.05^{c}	20.35±0.05^{d}	18.56±0.03^{e}	22.18±0.11^{a}	21.17±0.12^{c}	21.60±0.04^{b}
	**Texture**							
	Day 0	1284.74±104.18^{bc}	1584.69±141.60^{d}	2220.74±110.34^{b}	1435.25±77.01^{d}	1747.63±199.09^{c}	1266.15±62.22^{d}	2735.73±76.35^{a}
Hardness (gf)	Day 15	2696.99±92.60^{f}	4116.30±127.88^{de}	5519.49±315.17^{ab}	4955.98±251.42^{bc}	4517.81±198.09^{cd}	6102.46±648.56^{a}	5756.63±799.86^{a}
	Day 30	4351.14±287.97^{c}	5312.84±154.39^{b}	nm	nm	6390.99±546.5^{a}	nm	nm
	Day 0	0.91±0.01^{d}	0.88±0.02^{bc}	0.72±0.03^{b}	0.74±0.03^{b}	0.76±0.02^{b}	0.81±0.02^{a}	0.73±0.01^{b}
Springiness	Day 15	0.82±0.01^{a}	0.76±0.02^{bc}	0.79±0.02^{ab}	0.74±0.02^{c}	0.76±0.02^{bc}	0.75±0.02^{bc}	0.72±0.04^{c}
	Day 30	0.82±0.02^{a}	0.75±0.02^{b}	nm	nm	0.75±0.07^{b}	nm	nm
	Day 0	0.71±0.02^{d}	0.60±0.03^{ab}	0.50±0.01^{b}	0.51±0.06^{b}	0.55±0.01^{a}	0.52±0.01^{b}	0.51±0.02^{b}
Cohesiveness	Day 15	0.49±0.04^{bc}	0.40±0.01^{cde}	0.52±0.05^{b}	0.43±0.02^{bcd}	0.41±0.02^{cde}	0.49±0.12^{bc}	0.61±0.06^{a}
	Day 30	0.45±0.02^{a}	0.34±0.03^{b}	nm	nm	0.39±0.03^{ab}	nm	nm
Chewiness	Day 0	828.54±70.46^{c}	832.87±116.75^{c}	799.28±75.46^{b}	543.42±35.63^{c}	733.84±73.53^{b}	529.96±35.35^{c}	1012.27±21.87^{a}

		Day 15	1082.17±47.76^{d}	1236.76±49.36^{cd}	2249.46±203.31^{a}	1571.42±126.75^{b}	1419.52±36.41^{bc}	2225.28±352.04^{a}	2502.77±260.12^{a}
		Day 30	1610.31±156.28^{ab}	1348.65±126.58^{b}	nm	nm	1870.13±112.49^{a}	nm	nm
Resilience		Day 0	0.33±0.04^{e}	0.26±0.02^{ab}	0.19±0.05^{b}	0.21±0.06^{b}	0.22±0.01^{a}	0.22±0.01^{a}	0.20±0.01^{b}
		Day 15	0.179±0.02^{bcd}	0.16±0.04^{de}	0.20±0.02^{bc}	0.17±0.01^{cde}	0.16±0.01^{de}	0.22±0.05^{ab}	0.25±0.03^{a}
		Day 30	0.165±0.08^{a}	0.14±0.01^{a}	nm	nm	0.17±0.01^{a}	nm	nm
		**Color**							
Crust	L^*	Day 0	58.98±3.05^{c}	69.12±1.96^{ab}	56.17±3.52^{c}	63.45±4.34^{b}	58.18±2.81^{c}	73.17±1.31^{a}	68.15±2.00^{ab}
		Day 15	54.13±3.09^{e}	64.23±2.99^{ab}	55.11±3.62^{de}	65.85±3.14^{a}	57.18±2.91^{de}	65.03±2.18^{ab}	65.36±3.62^{ab}
		Day 30	56.11±4.62^{b}	66.14±3.76^{a}	55.82±2.67^{b}	65.16±2.98^{a}	61.75±3.66^{ab}	65.21±2.22^{a}	66.66±3.38^{a}
	a^*	Day 0	12.47±1.88^{a}	3.21±1.83^{c}	8.80±2.31^{b}	2.82±0.78^{d}	4.48±0.36^{c}	1.64±0.85^{e}	0.38±0.87^{f}
		Day 15	14.10±1.08^{a}	6.72±1.96^{cd}	10.77±0.84^{b}	3.29±2.49^{e}	5.35±2.08^{cde}	4.51±2.12^{de}	3.51±1.57^{e}
		Day 30	12.24±0.62^{a}	4.55±2.11^{b}	10.01±0.97^{a}	3.79±2.05^{b}	4.58±1.21^{b}	3.64±1.58^{b}	2.04±1.17^{b}
	b^*	Day 0	30.99±2.86^{a}	25.92±2.255^{b}	24.16±1.03^{b}	16.58±1.80^{d}	12.15±1.16^{e}	21.29±1.24^{c}	17.01±2.03^{d}
		Day 15	30.32±2.89^{a}	29.49±1.55^{ab}	27.56±2.36^{ab}	24.22±3.80^{cd}	21.25±3.32^{e}	27.56±2.44^{ab}	26.71±1.62^{bc}
		Day 30	33.28±2.65^{a}	28.21±2.03^{b}	26.28±1.07^{bc}	23.67±3.09^{cd}	19.87±2.32^{d}	25.69±2.59^{bc}	23.26±1.16^{cd}
Crumb	L^*	Day 0	75.03±0.84^{a}	71.43±1.24^{b}	66.43±0.91^{c}	61.26±1.27^{d}	59.45±0.66^{d}	69.67±1.76^{b}	64.39±1.59^{c}
		Day 15	75.97±1.74^{a}	71.31±1.14^{bcd}	66.97±1.31^{f}	62.81±1.79^{g}	61.60±0.85^{g}	71.84±0.99^{bc}	65.48±2.07^{f}
		Day 30	73.75±2.50^{a}	67.91±1.70^{b}	65.32±1.22^{bc}	64.17±1.79^{c}	59.96±2.13^{d}	71.64±0.80^{a}	65.40±1.40^{bc}
	a^*	Day 0	-2.27±0.29^{f}	-0.99±0.16^{e}	0.44±0.43^{c}	1.97±0.29^{b}	3.68±0.14^{a}	-0.45±0.30^{d}	-0.03±0.42^{cd}
		Day 15	-0.99±0.43^{de}	-0.89±0.22^{de}	0.86±0.43^{b}	1.20±0.58^{b}	3.11±0.16^{a}	-0.72±0.22^{cd}	-0.62±0.48^{cd}
		Day 30	-0.76±0.78^{c}	-0.43±0.76^{c}	1.59±0.75^{b}	1.12±0.69^{b}	3.37±0.21^{a}	-0.27±0.14^{c}	-0.21±0.25^{c}
	b^*	Day 0	24.32±0.80^{a}	16.48±1.10^{b}	16.85±0.39^{b}	13.82±0.37^{c}	10.56±0.18^{d}	17.26±0.18^{b}	14.67±1.36^{c}
		Day 15	24.24±0.55^{a}	19.78±0.49^{b}	19.25±0.58^{bc}	15.68±0.74^{f}	15.06±0.49^{f}	18.14±0.28^{e}	15.07±0.42^{f}
		Day 30	23.51±0.96^{a}	19.55±0.57^{b}	18.00±0.92^{c}	14.89±0.46^{d}	13.71±0.66^{d}	17.69±0.37^{c}	14.71±1.30^{d}

Results are displayed as the means ± standard deviation. Means followed by the different letter within a line are significantly different ($p<0.05$).). CC: Control muffin, AC: Aquafaba muffin, BC: Banana muffin, FC: Flaxseed muffin, PC: Psyllium muffin, SWC: Soapwort muffin, CHC: Chia muffin. nm: not measurable

Supplementary Table S2. Microbiological analysis results of muffin samples throughout shelf life

	Aerobic Plate Count (APC), CFU/g			*E. coli*, CFU/g			Coliforms, CFU/g			*S. aureus*, CFU/g			Yeasts and Molds, CFU/g			*Salmonella* spp., CFU/g		
	Day 0	Day 15	Day 30	Day 0	Day 15	Day 30	Day 0	Day 15	Day 30	Day 0	Day 15	Day 30	Day 0	Day 15	Day 30	Day 0	Day 15	Day 30
**CC**	$2.7\text{x}10^{2\ \text{d,B}}$	$7.5\text{x}10^{2\ \text{c,A}}$	$2.6\text{x}10^{2\ \text{g,B}}$	<10	<10	<10	<10	<10	<10	<10	<10	<10	<10	<10	<10	<LOD	<LOD	<LOD
**AC**	$5.1\text{x}10^{2\ \text{c,C}}$	$1.3\text{x}10^{3\ \text{b,B}}$	$2.1\text{x}10^{3\ \text{a,A}}$	<10	<10	<10	<10	<10	<10	<10	<10	<10	<10	<10	<10	<LOD	<LOD	<LOD
**BC**	$7.0\text{x}10^{2\ \text{b,B}}$	$1.9\text{x}10^{3\ \text{a,A}}$	$1.7\text{x}10^{3\ \text{b,A}}$	<10	<10	<10	<10	<10	<10	<10	<10	<10	<10	<10	<10	<LOD	<LOD	<LOD
**FC**	$2.9\text{x}10^{2\ \text{d,B}}$	$5.9\text{x}10^{2\ \text{d,A}}$	$6.3\text{x}10^{2\ \text{e,A}}$	<10	<10	<10	<10	<10	<10	<10	<10	<10	<10	<10	<10	<LOD	<LOD	<LOD
**PC**	$2.2\text{x}10^{2\ \text{e,C}}$	$5.7\text{x}10^{2\ \text{d,A}}$	$4.8\text{x}10^{2\ \text{f,B}}$	<10	<10	<10	<10	<10	<10	<10	<10	<10	<10	<10	<10	<LOD	<LOD	<LOD
**SWC**	$2.5\text{x}10^{2\ \text{e,B}}$	$7.4\text{x}10^{2\ \text{c,A}}$	$7.1\text{x}10^{2\ \text{d,A}}$	<10	<10	<10	<10	<10	<10	<10	<10	<10	<10	<10	<10	<LOD	<LOD	<LOD
**CHC**	$8.9\text{x}10^{2\ \text{a,C}}$	$2.1\text{x}10^{3\ \text{e,A}}$	$9.6\text{x}10^{2\ \text{c,B}}$	<10	<10	<10	<10	<10	<10	<10	<10	<10	<10	<10	<10	<LOD	<LOD	<LOD

Results are displayed as the means ± standard deviation. Different lower case letters in the same column indicate the difference between samples. Different capital letters on the same row indicate the difference between days (P<0.05). CC: Control muffin, AC: Aquafaba muffin, BC: Banana muffin, FC: Flaxseed muffin, PC: Psyllium muffin, SWC: Soapwort muffin, CHC: Chia muffin. LOD: limit of detection; for *Salmonella* spp., <1 CFU/25 g

Supplementary Table S3. Explained variance of the first seven principal components

Variables	PC1	PC2	PC3	PC4	PC5	PC6	PC7
K'	-0.181	-0.319	-0.900	-0.124	0.062	0.175	-0.045
n'	-0.109	-0.131	-0.095	0.070	-0.757	0.032	0.258
R2'	-0.481	0.276	-0.163	0.363	0.628	0.356	0.066
K"	-0.381	-0.207	-0.860	-0.163	0.123	0.160	-0.047
n"	0.113	-0.144	0.600	0.171	0.148	0.015	-0.312
R2"	-0.652	-0.203	-0.107	0.427	0.394	0.423	0.041
Baking Loss	0.754	-0.246	-0.212	-0.189	-0.356	-0.128	-0.096
VI	0.227	0.530	0.584	0.379	-0.177	-0.314	0.009
SI	0.645	0.131	0.486	0.072	0.064	0.253	0.096
UI	-0.004	-0.005	0.112	0.242	0.056	-0.053	0.839
Hardness	-0.489	-0.757	-0.348	-0.184	0.021	0.007	0.029
Springiness	0.129	0.252	0.775	0.440	0.273	-0.106	0.009
Cohesiveness	-0.055	0.213	0.548	0.774	0.021	-0.158	0.070
Chewiness	-0.504	-0.613	0.155	0.392	0.083	-0.114	0.234
Resilience	-0.011	0.290	0.582	0.681	-0.086	-0.186	0.037
Crust-L*	0.597	-0.354	0.505	-0.365	0.148	0.006	-0.065
Crust-a*	-0.579	0.514	0.155	0.525	-0.251	-0.075	-0.052
Crust-b*	-0.441	0.278	0.741	0.278	-0.081	0.214	-0.073
Crumb-L*	-0.242	0.127	0.925	0.114	-0.087	-0.021	0.109
Crumb-a*	0.255	0.095	-0.916	-0.126	0.198	-0.151	-0.019
Crumb-b*	-0.303	0.209	0.735	0.328	-0.440	0.092	0.004
Moisture	0.505	0.099	-0.099	-0.630	-0.436	-0.355	-0.066
Ash	-0.174	-0.398	-0.579	-0.476	-0.257	0.407	-0.119
Protein	-0.094	0.081	0.370	0.813	-0.428	0.004	-0.012
Total Carbohydrate	-0.475	-0.077	-0.123	-0.304	0.762	0.193	0.075
TDF	0.723	-0.340	-0.438	-0.117	-0.060	-0.103	0.070
Fat	-0.070	0.095	0.262	0.817	-0.331	0.232	0.033
Energy	-0.308	0.077	0.132	0.602	0.107	0.283	0.155
P	-0.266	-0.237	0.099	0.807	-0.440	-0.003	0.005
Na	-0.131	0.076	0.313	0.883	0.112	-0.220	0.124
Mg	0.091	-0.706	-0.341	-0.101	0.224	0.558	-0.019
K	-0.791	-0.023	-0.047	-0.103	0.506	0.313	0.038
Ca	0.148	-0.956	-0.092	0.217	0.014	0.058	0.034
TPC-Undigested	-0.321	0.483	0.643	0.162	0.077	0.119	0.096
TPC-Gastric	0.511	0.146	-0.340	0.151	0.003	0.738	-0.098
TPC-Intestinal	-0.006	-0.412	-0.022	0.856	-0.291	-0.088	0.023
CUPRAC-Undigested	-0.080	0.025	-0.387	-0.308	0.803	-0.012	0.119
CUPRAC-Gastric	-0.321	0.085	0.928	0.056	0.108	0.075	0.053
CUPRAC-Intestinal	-0.291	-0.070	0.755	-0.023	0.559	0.068	0.109
DPPH-Undigested	-0.350	0.526	-0.587	-0.433	-0.028	0.228	-0.087
DPPH-Gastric	-0.422	0.400	0.405	-0.328	0.192	0.575	-0.016
DPPH-Intestinal	0.479	-0.407	-0.183	0.116	-0.418	-0.613	0.008

Aspartic Acid	0.421	0.629	0.289	-0.153	-0.145	0.521	-0.080
Glutamic Acid	0.841	0.131	-0.069	-0.341	0.376	0.026	0.052
Asparagine	0.771	0.442	0.101	0.229	0.157	0.256	-0.019
Serine	0.021	-0.049	0.457	0.078	0.852	0.180	0.090
Glutamine	0.586	-0.117	-0.492	-0.331	0.056	0.080	0.274
Histidine	0.602	0.453	0.132	0.480	-0.243	0.087	-0.218
Glycine	0.832	0.366	-0.266	-0.146	0.207	-0.033	0.112
Threonine	0.900	0.210	-0.149	-0.183	0.127	-0.127	0.048
Arginine	0.914	0.258	-0.150	-0.084	-0.116	0.166	-0.044
Alanine	0.809	0.467	-0.066	0.183	0.036	0.244	-0.060
Tyrosine	0.931	0.124	-0.178	-0.184	-0.137	-0.046	-0.085
Cystine	-0.047	0.239	-0.134	0.170	-0.937	-0.060	-0.042
Valine	0.860	0.252	-0.050	-0.355	-0.005	0.137	-0.149
Methionine	0.014	0.949	0.153	0.227	-0.131	-0.007	-0.012
Norvaline	-0.054	-0.877	-0.255	0.142	-0.187	-0.306	0.005
Trptophan	0.168	-0.903	-0.246	-0.262	0.052	0.059	-0.016
Phenylalanine	0.976	0.105	0.070	0.030	0.003	-0.156	0.014
Isoleucine	0.907	-0.368	0.085	0.061	0.012	-0.117	0.034
Leucine	0.542	0.830	0.064	-0.011	0.053	0.007	0.016
Lysine	0.341	0.930	0.032	0.095	-0.003	0.032	0.004
Hdroxyproline	0.317	-0.085	-0.315	-0.156	0.759	-0.395	0.098
Sarcosine	0.306	0.859	0.056	-0.105	-0.013	0.215	0.149
Proline	-0.082	-0.991	0.062	0.004	-0.041	0.012	0.018
